# A Simple Zinc-Mediated Method for Selenium Addition to Michael Acceptors

**DOI:** 10.3390/molecules25092018

**Published:** 2020-04-26

**Authors:** Francesca Giulia Nacca, Bonifacio Monti, Eder João Lenardão, Paul Evans, Claudio Santi

**Affiliations:** 1 Group of Catalysis, Synthesis and Organic Green Chemistry, Department of Pharmaceutical Sciences University of Perugia Via del Liceo 1, 06123 Perugia, Italy; nacca.francescagiulia@gmail.com (F.G.N.); bonifaciomonti@gmail.com (B.M.); 2 Centre for Synthesis and Chemical Biology, School of Chemistry University College Dublin, Dublin D04, N2E5, Ireland; paul.evans@ucd.ie; 3 LASOL–CCQFA, Universidade Federal de Pelotas—UFPel, P.O. Box 354, 96010-900 Pelotas, RS, Brazil; lenardao@ufpel.edu.br

**Keywords:** zinc, selenium, nucleophilic addition, seleno-Michael reaction, conjugate addition, reduction

## Abstract

In this work, we focused our attention on seleno-Michael type reactions. These were performed using zinc-selenolates generated in situ from diphenyl diselenide **1**, 1,2-bis(3-phenylpropyl)diselenide **30**, and protected selenocystine **31** via an efficient biphasic Zn/HCl-based reducing system. Alkenes with a variety of electron-withdrawing groups were investigated in order to gauge the scope and limitations of the process. Results demonstrated that the addition to acyclic α,β-unsaturated ketones, aldehydes, esters amides, and acids was effectively achieved and that alkyl substituents at the reactive β-centre can be accommodated. Similarly, cyclic enones undergo efficient Se-addition and the corresponding adducts were isolated in moderate to good yield. Vinyl sulfones, α,β-unsaturated nitriles, and chalcones are not compatible with these reaction conditions. A recycling experiment demonstrated that the unreacted Zn/HCl reducing system can be effectively reused for seven reaction cycles (91% conversion yield at the 7° recycling rounds).

## 1. Introduction

Organoselenium derivatives are widely utilized in the area of organic chemistry, this is due to their great versatility. The introduction of an organoselenium moiety can be easily obtained starting from diselenides that can be readily converted into electrophilic, nucleophilic, or radical species through oxidative, reductive, or homolytic cleavage of the selenium–selenium bond [1,2,3,4,5,6,7,8,9]. Researchers have taken advantage of this chemistry to synthesize a range of different bioactive compounds incorporating selenium [10,11,12]. Some selenium-containing molecules can behave as antioxidants, enzyme mimics and inhibitors, immunomodulators, cytoprotectors, antitumoral, anti-inflammatory, antihypertensive, and anti-infective agents [13,14,15,16]. Additionally, we have reported on the immunomodulatory and cytotoxic properties of selenium-containing compounds and their activity as inhibitors of several different enzymes and proteins, such as glutathione transferase (GSTp) and NCp7, which indicate interesting potential applications in anticancer and anti-HIV chemotherapy [17,18].

Nucleophilic selenolates are often generated in situ by reduction of the selenium–selenium bond in diselenides, or by the insertion of elemental selenium into organometallic species, such as Grignard reagents or organolithium derivatives [19,20,21]. The main drawbacks of these procedures are the use of inert conditions and the instability of the desired selenolates, which can rapidly oxidize to the corresponding diselenides. A variety of low-valent metals have been used for reductive cleavage of the Se–Se bond: Cd(0) [22], La(0) [23], In(0) [24], In(I) [25], Sm(0) [26,27], Sm(II) [28], Sn(0) [29], Yb(II) [30], Cu(II)/Sn(II) [31]. In addition, several different reducing agents, such as: NaBH_4_ [32], LiAlH_4_ [33], LiEt_3_BH [34], DIBAL [35], NaB(OMe)_3_H [36], H_3_PO_2_ [37], N_2_H_4_ [38], or Rongalite have all been used to good effect [39].

**Scheme 1:**
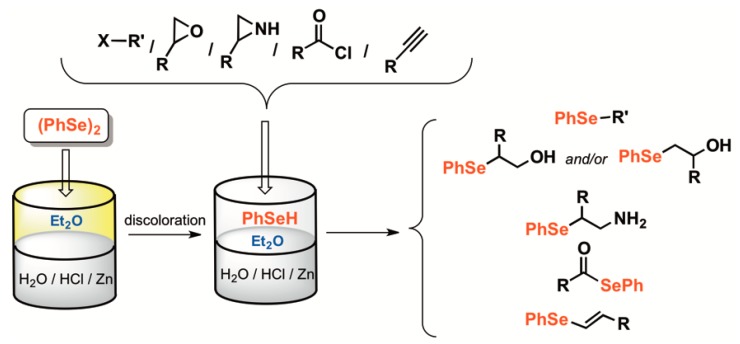
One-pot zinc-mediated biphasic diselenide reduction-selenylation reaction. 1^st^ step: diselenide reduction evidenced by discoloration; 2^nd^ step selenylation (substitution and addition reactions).

In order to obtain nucleophilic selenium species, we became interested in the applicability of zinc. The use of this metal in the S–S and Se–Se bond cleavages was already described, in combination with Lewis acids, such as aluminum chloride (AlCl_3_) [40,41], zirconium tetrachloride (ZrCl_4_) [42], RuCl_3_ [43] and TiCl_4_ [44], or simply basic [45] or acidic conditions [46]. The zinc insertion into Se-Halogen bond leading to the umpolung of the selenium atom and the formation of the first class of bench stable zinc selenolates, was deeply investigated, with a broad synthetic applicability [47,48,49,50,51,52,53,54,55]. Previously, it has been reported that this diselenide zinc-mediated reduction can be successfully coupled with chemistry aimed at derivatizing the sensitive, in situ formed selenol [56]. For instance, various epoxides [56], aziridines [57], alkyl and acyl halides [56,58] can engage in nucleophilic substitution processes and generate the corresponding organoselenide products in good to excellent yields (Scheme 1). A feature of this approach is that using a biphasic system (Et_2_O/HCl(aq)), the product partitions into the organic phase, facilitating the separation and the reuse of the unreacted zinc, as well as of the aqueous acidic medium. Indeed, often a simple solvent removal leads to the isolation of Se-derivatives without the requirement for additional purification. This method proved to be the best alternative to reduce S–S and Se–Se bonds of some non-natural peptides, as recently demonstrated by Flemer [59,60].

**Scheme 2:**
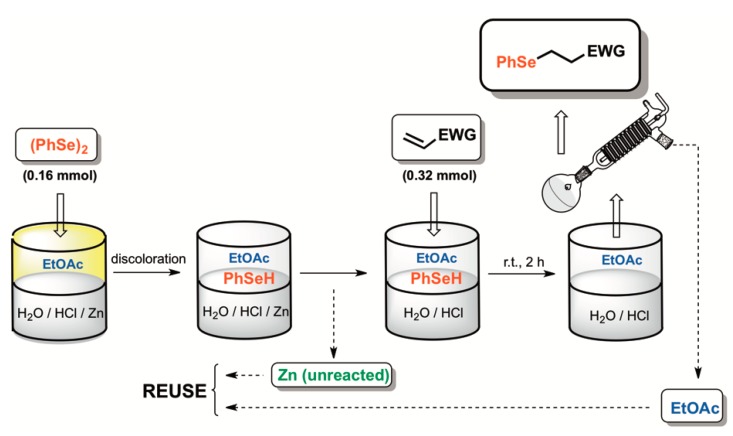
One-pot zinc-mediated biphasic diselenide reduction-Se-conjugate addition reaction.

More recently, we have demonstrated that the soft-selenium-based nucleophile can efficiently add to a range of alkynes [61] and we collected some of these data in a recently published review article [62]. As an extension of these studies, in this current work, we report that this *in situ* formed a nucleophilic selenenylating mixture that can efficiently react with a range of Michael acceptors (Scheme 2).

## 2. Results and Discussion

Considering that conjugated systems, mainly aldehydes and ketones (EWG = CHO, COR) can be prone to reduction in the presence of Zn/HCl [63], new conditions were optimized with the removal of unreacted zinc after the discoloration of the organic phase, and before the addition of the substrates. Furthermore, to improve the “greenness” of the overall procedure, the organic phase (diethyl ether) was changed to ethyl acetate, because this solvent is easier to recycle, has a lower vapor pressure, and presents a series of other aspects (in terms of health and environmental impact), which have given it a recommended ranking by the CHEM21 selection guide [64].

Initially, a range of monosubstituted α,β-unsaturated alkenes presenting a single electron-withdrawing group were considered. As precedingly reported [56,58], the nucleophilic selenenylating mixture arises from the reduction of the diselenide through the oxidative insertion of zinc into the Se–Se bond affording the *in situ* formation of a selenolate [PhSeZnSePh] that, in the acidic biphasic system, is in equilibrium with the corresponding selenol. This process takes roughly 20 min, and experimentally the reduction progress can be visually determined by the loss of the yellow coloration in the organic phase, caused by diphenyl diselenide. The mixture was then decanted in order to remove residual unreacted zinc (used in excess) and the desired alkene was added. Following this protocol, a range of differently substituted alkenes underwent efficient conjugate addition and the adducts were isolated in moderate to good yield after stirring for 2 h (Table 1).

As shown in Table 1, Entry 1, methyl vinyl ketone **2** gave the adduct **11** in excellent yield. Similarly, acrolein **3**, *trans-*but-2-enal **4**, and *trans-*pent-2-enal **5** gave the corresponding phenylselenide adducts **12**–**14** in moderate to good yields (Table 1, Entries 2–4). The low yield in the case of 15 was primarily due to the limited stability of this Se-adduct during purification using silica-gel chromatography and not to a low conversion of the starting material. Next, we turned our attention to carboxylic acids and their derivatives. Methyl acrylate **7** and acrylic acid **8** gave ester **16** and acid **17** in good yields (Table 1, Entries 6 and 7). In the case of unsaturated amide **9** (Table 1, Entry 8), a low yield of Se-adduct **18** was isolated presumably due to poor conversion stemming from the reduced electrophilicity of the Michael-acceptor. Finally, under the conditions developed, pulegone **10** gave the adduct **19** in excellent yield as a mixture of diastereomers at the new chiral center (Table 1, Entry 9). Less electron-poor alkenes, such as phenyl vinyl sulfone, acrylonitrile, and chalcones did not prove to be suitable substrates and no conversions were observed under the conditions optimized for substrates **2**–**10**.

The reaction between diphenyl diselenide **1** and a range of cyclic enones **20**–**24** was next considered (Table 2). As observed in Table 2, Entry 1, using cyclopentenone **20**, the adduct **25** was isolated in excellent yield. Similarly, cyclohexenone **21** and cycloheptenone **22** gave the corresponding Se-adducts **26** and **27** in comparable yields (Table 2, Entries 2 and 3). The substituent in the γ-position in cyclopentenone **23** was compatible with this process and product **28** was isolated as a 75:25 mixture of undetermined diastereomers (Table 2, Entry 4). Finally, the natural product jacaranone **24**, bearing two-potential sites for nucleophilic attack, was found to form in low yield (29%) mono-adduct **29** as a single undetermined diastereomer under the optimized conditions (Table 2, Entry 5).

Finally, the possibility of using alternative diselenides in the reaction with enones was considered. As shown in Table 3, both 1,2-bis(3-phenylpropyl)diselenide **30** and *N,O*-protected selenocystine **31** proved to generate the corresponding adducts in moderate to good yields.

As shown in Table 3, Entries 1–2, Se-adducts **34**–**35**, obtained starting from 1,2-bis(3-phenylpropyl)diselenide **30**, were isolated in good yields whereas the reactivity of the nucleophilic selenenylating mixture prepared starting from the *N,O*-protected selenocystine **31** is appreciably reduced affording the target compounds **35**, **36,** and **37** only in moderate yields (Entries 3, 4, and 5). Nevertheless, despite the reduced reactivity, it is worth mentioning that in these latter cases the Boc protecting group was not unduly affected by the acidic media. In the cases in which the conjugate addition of the organoselenium moiety generates a new chiral center in the presence of a preformed one in the substrate and or in the reagent (Entries 2, 4, and 5), a moderated stereoselectivity was obtained. In these cases, the reaction afforded a mixture of diastereomers from which, only in one case, was it possible to isolate the major one as a stereochemically undetermined product **34**. In the other two cases, probably due to their high polarity, it was not possible to separate the diastereomers **36** and **37** by flash chromatography (Table 3, Entries 4–6, respectively).

**Scheme 3:**
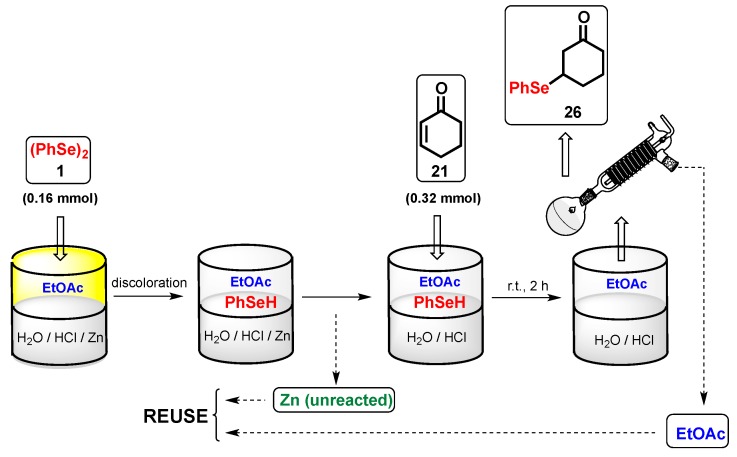
Reuse of the aqueous phase in the Se-conjugate addition of diphenyl diselenide **1** to cyclohexenone **21.**

**Figure 1:**
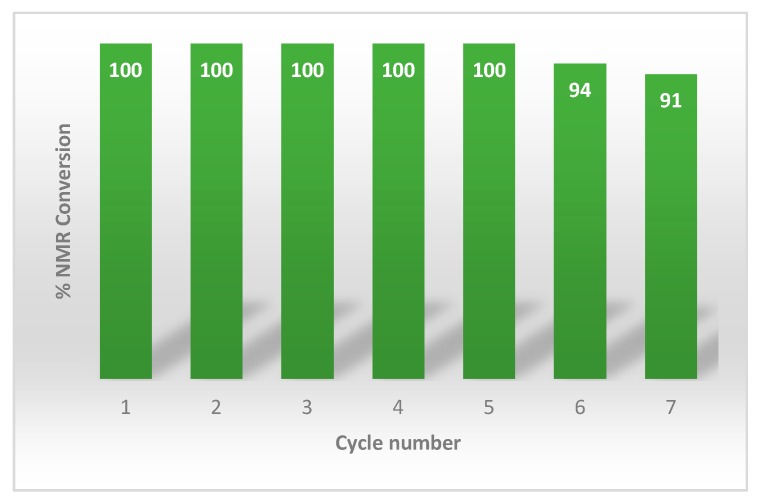
NMR conversion in the first seven reuses of the aqueous phase in the Se-conjugate addition of diphenyl diselenide **1** to cyclohexenone **21**.

The recyclability of the biphasic medium and of the unreacted zinc was investigated in the reaction of diphenyl diselenide **1** with cyclohexanone **21** (in a 0.04 M solution of HCl_10%*v*/*v*_/EtOAc) as a general model (Scheme 3). After the reaction, the superior organic phase containing the adduct **26** was separated, and part of the organic solvent was recovered by distillation. The hydrochloric aqueous solution and the unreacted zinc were reused for a subsequent cycle, by the addition of fresh EtOAc, PhSeSePh **1**, and after discoloration, a new amount of cyclohexanone **21** was added. As shown in Figure 1, the conversion for the first five cycles (evaluated by ^1^H-NMR spectroscopy) was found to be quantitative and it only decreased, slightly, following the 6^th^ and 7^th^ cycles.

## 3. Materials and Methods

Reactions were conducted in closed vials (6 mL) and were stirred with a Teflon-coated magnetic stirring bar. Solvents and reagents were used as received. The analytical thin layer chromatography (TLC) was performed on silica gel 60 F254 precoated aluminum foil sheets (Merck, Darmstadt, Germany) and visualized by UV irradiation (Spectroline^®^ UV light, Sigma-Aldrich, St Louis, Missouri, USA) or by use of a KMnO_4_ stain (Merck, Darmstadt, Germany) Silica gel Kiesinger 60 (70–230 mesh) was used for flash column chromatography. NMR spectroscopic (Bruker, Fällanden, Switzerland) experiments were obtained at 25 °C on Bruker DPX spectrometers operating at the specified frequencies. ^1^H and ^13^C chemical shifts (δ) are reported in parts per million (ppm) and they are relative to TMS 0.0 ppm and/or the solvent peak of CDCl_3_ at δ 7.26 and δ 77.00 ppm in ^1^H and ^13^C NMR spectra, respectively. Data are reported as follows: Chemical shift (multiplicity, coupling constants, number of hydrogen atoms, where applicable, and assignment where possible). Abbreviations are as follows: S (singlet), d (doublet), t (triplet), q (quartet), p (pentet), dd (doublet of doublet), ddd (doublet of doublet of doublet), dddd (doublet of doublet of doublet of doublet), dt (doublet of triplet), ddt (doublet of doublet of triplet), tt (triplet of triplet), m (multiplet), and bs (broad signal). Coupling constants (*J*) are quoted in Hertz (Hz) to the nearest 0.1 Hz. High resolution mass spectra were carried out on a VG analytical 70-E mass spectrometer (VG Analytical Ltd., Manchester, UK) under an electrospray ionization condition (ESI). IR spectra were recorded on a Bruker Alpha FTIR spectrometer (Billerica, MA, USA). Optical rotation measurements were recorded using a Perkin-Elmer Model 343 polarimeter at 589 nm (Perkin Elmer’s, Waltham, MA, USA) and are given in units of 10^−1^degcm^2^g^−1^. Melting points are uncorrected and were recorded using a Gallenkamp electrothermal melting point apparatus (Cole-Parmer Ltd., Staffordshire, UK). Alkenes **2**–**10** and **20**–**22** are commercially available and were used without further purification; alkenes **23** and **24** (with OTBS and NHBoc substituents) were synthesized as described [67,68]. Jacaranone **25** was prepared by oxidation of the methyl 4-hydroxyphenylacetic acid.

Diphenyl diselenide **1** is commercially available, whereas diselenide **32** was prepared according to the procedure reported in the literature [69]. Selenocystine is commercially available and it was *N*- and *O*-protected, according to the procedure reported in the literature [70] in order to obtain compound **33**.

### General Procedure for the Se-Michael-Type Addition

Diselenides (**1**, **32,** and **33**) (0.16 mmol) were introduced to a biphasic system composed of EtOAc (2 mL) and HCl (10% *v*/*v*, 2 mL). Then, 1.6 mmol (10 equiv.) of zinc powder (or turnings) were added. The vial was closed and the mixture was vigorously stirred (approx. 800 rpm) at room temperature until the discoloration of the organic layer (approx. 20 min). After that, zinc was removed and the olefin (0.32 mmol) was added to the liquid. The reaction was stirred at room temperature for an additional 2 h. The ethyl acetate was separated and the resultant aqueous phase extracted with EtOAc (3 × 2 mL). The organic layers were combined, washed with brine, dried over Na_2_SO_4_, filtered, and the solvent was removed under reduced pressure.

In the recycling, experiment diselenide **1** (0.16 mmol) was introduced to a biphasic system composed of EtOAc (4 mL) and HCl (10% *v*/*v*, 4 mL). Then, 1.6 mmol (10 equiv.) of zinc turnings were added. The vial was closed and the mixture was vigorously stirred (approx. 800 rpm) at room temperature until the discoloration of the organic layer (approx. 20 min). After that, zinc was removed and recovered and 0.32 mmol of **21** was added to the liquid. The reaction was stirred at room temperature for an additional 2 h. The ethyl acetate was separated from the aqueous layer that was recovered. The organic phase was distilled and partially recovered to obtain a crude that was analyzed by proton NMR for the conversion yield. Recovered acidic water, EtOAc, and unreacted zinc mixed with small amounts of fresh solvents to reach the original volumes (4 + 4 mL) were reused for seven subsequent cycles.

*4-(Phenylselanyl)butan-2-one* (**11**) [71]: Isolated as a yellow oil in 91% yield (0.066 g) without purification. ^1^H NMR (200 MHz, CDCl_3_) δ: 7.60–7.45 (m, 2 H, CH), 7.30–7.20 (m, 3 H, CH), 3.02 (t, *J* = 6.7 Hz, 2 H, CH_2_), 2.80 (t, *J* = 6.7 Hz, 2 H, CH_2_), 2.18 (s, 3 H, CH_3_) ppm; ^13^C NMR (50.31 MHz, CDCl_3_) δ = 207.1, 132.7, 129.6, 129.1, 127.0, 44.0, 29.9, 20.4 ppm.

*3-(Phenylselanyl)propanal* (**12**) [72]: Isolated as a yellow oil in 44% yield (0.030 g) after flash column chromatography, eluent cyclohexane/ethyl acetate (9:1). ^1^H NMR (500 MHz, CDCl_3_) δ: 9.74 (bs, 1 H, CH), 7.51–7.48 (m, 2 H, CH), 7.29–7.25 (m, 3 H, CH), 3.09 (t, 2 H, *J* = 7.1 Hz, CH_2_), 2.85 (dt, 2 H, *J* = 0.9 and 7.1 Hz, CH_2_) ppm; ^13^C-NMR (125.77 MHz, CDCl_3_) δ = 200.6, 133.3, 129.2, 129.1, 127.4, 44.2, 18.9 ppm.

*3-(Phenylselanyl)butanal* (**13**) [73]: Isolated as a yellow oil in 70% yield (0.051 g) after flash column chromatography, eluent petroleum ether/ethyl acetate (95:5).^1^H NMR (200 MHz, CDCl_3_) δ = 9.67–9.66 (m, 1 H, CH), 7.60–7.45 (m, 2 H, CH), 7.30–7.20 (m, 3 H, CH), 3.65 (sextet, *J* = 7.0 Hz, 1 H, CH), 2.73 (ddd, *J* = 1.8, 7.0, and 13.5 Hz, 1 H, CH_2_), 2.63 (ddd, *J* = 1.7, 7.0, and 13.5 Hz, 1 H, CH_2_), 1.39 (d, *J* = 7.0 Hz, 3 H, CH_3_) ppm; ^13^C NMR (50.31 MHz, CDCl_3_) δ = 200.8, 135.6, 129.2, 128.2, 128.0, 51.0, 31.7, 22.1 ppm.

*3-(Phenylselanyl)pentanal* (**14**) [74]: Isolated as a yellow oil in 60% yield (0.030 g) after flash column chromatography, eluent petroleum ether/ethyl acetate (95:5). ^1^H NMR (200 MHz, CDCl_3_) δ = 9.74–9.72 (m, 1 H, CH), 7.60–7.45 (m, 2 H, CH), 7.30–7.20 (m, 3 H, CH), 3.50 (p, *J* = 6.8 Hz, 1 H, CH), 2.78–2.71 (m, 2 H, CH_2_), 1.76–1.61 (m, 2 H, CH_2_), 1.10 (t, *J* = 7.3 Hz, 3 H, CH_3_) ppm; ^13^C NMR (50.31 MHz, CDCl_3_) δ = 201.0, 135.6, 129.0, 128.0, 127.6, 48.8, 40.0, 28.4, 12.3 ppm.

*3-Methyl-3-(phenylselanyl)butanal* (**15**) [74]: Isolated as a yellow oil in 22% yield (0.017 g) due to the limited stability during silica gel column chromatography, eluent cyclohexane/ethyl acetate (9:1). ^1^H NMR (400 MHz, CDCl_3_) δ = 9.86 (t, *J* = 2.7 Hz, 1 H, CH), 7.67–7.55 (m, 2 H, CH), 7.41–7.29 (m, 3 H, CH), 2.55 (d, *J* = 2.7 Hz, 2 H, CH_2_), 1.51 (s, 6 H, CH_3_) ppm. ^13^C NMR (100 MHz, CDCl_3_) δ = 201.8, 138.3, 129.1, 129.0, 126.9, 55.1, 41.9, 30.0 ppm.

*Methyl 3-(phenylselanyl)propanoate* (**16**) [44]: Isolated as a yellow oil in 79% yield (0.097 g) as pure compound without purification. ^1^H NMR (200 MHz, CDCl_3_) δ = 7.60–7.45 (m, 2 H, CH), 7.30–7.20 (m, 3 H, CH), 3.72 (s, 3 H, CH_3_), 3.15 (t, 2 H, *J* = 6.5 Hz, CH_2_), 2.74 (t, 2 H, *J* = 6.5 Hz, CH_2_) ppm; ^13^C NMR (50.31 MHz, CDCl_3_) δ = 172.6, 133.3, 131.5, 129.2, 127.3, 51.8, 35.1, 21.8 ppm.

*3-(Phenylselanyl)propanoic acid* (**17**) [75]: Isolated as a yellow oil in 60% yield (0.140 g) and purified by crystallization from petroleum ether and ethyl acetate. ^1^H NMR (200 MHz, CDCl_3_) δ = 7.66–7.56 (m, 2 H, CH), 7.36–7.25 (m, 3 H, CH), 3.18 (t, 2 H, *J* = 7.0 Hz, CH_2_), 2.86 (t, 2 H, *J* = 7.0 Hz, CH_2_) ppm; ^13^C-NMR (CDCl_3_, 50.31 MHz): δ = 178.6, 133.3, 131.3, 129.1, 127.3, 35.0, 21.5 ppm.

*N,N-Dimethyl-3-(phenylselanyl)propanamide* (**18**): Isolated as a yellow oil in 25% yield (0.02 g) after flash column chromatography, eluent cyclohexane/ethyl acetate (1:1). R*_f_* = 0.29 (c-Hex/EtOAc 1:1); ν_max_ = 3000, 2926, 1642, 1578, 1477, 1437, 1397, 1326, 1303, 1264, 1189, 1128, 1072, 1022 cm^−1^. ^1^H NMR (500 MHz, CDCl_3_) δ = 7.60–7.45 (m, 2 H, CH), 7.30–7.20 (m, 3 H, CH), 3.18 (t, 2 H, *J* = 7.6 Hz, CH_2_), 2.94 (s, 3 H, CH_3_), 2.92 (s, 3 H, CH_3_), 2.73 (t, 2 H, *J* = 7.6 Hz, CH_2_) ppm; ^13^C NMR (125.77 MHz, CDCl_3_) δ = 171.4, 132.6, 130.1, 129.0, 126.9, 37.0, 35.4, 34.2, 22.4 ppm. HRMS (ESI^+^) C_11_H_15_NONaSe (MNa^+^) calcd: 280.0217; found: 280.0230.

*(R)-5-Methyl-2(R/S)-(2-(phenylselanyl)propan-2-yl)cyclohexanone* (**19**): Isolated as a yellow oil and a mixture of two isomers (62/38) in 95% yield (0.031 g) after flash column chromatography, eluent petroleum ether/DCM 20:80. ν_max_ = 3070, 3056, 2954, 2925, 2869, 1708, 1576, 1474, 1455, 1437, 1379, 1362, 1285, 1208, 1117, 1088, 1046, 1021 cm^−1^. For ^1^H NMR (400 MHz, CDCl_3_) and ^13^C NMR (100 MHz, CDCl_3_) of the mixture see Supplementary Materials. HRMS (ESI ^+^) C_16_H_22_ONaSe (MNa^+^) calcd: 333.0735; found: 333.0734.

*3-(Phenylselanyl)cyclopentanone* (**25**) [76]: Isolated as a yellow oil in 93% yield (0.072 g) without purification. ^1^H NMR (200 MHz, CDCl_3_) δ = 7.70–7.50 (m, 2 H, CH), 7.30–7.20 (m, 3 H, CH), 3.89 (m, 1 H, CH), 2.70 (dd, *J* = 7.3 and 17.8 Hz, 1 H, CH_2_), 2.50–1.75 (m, 5 H, CH_2_) ppm; ^13^C NMR (50.31 MHz, CDCl_3_) δ = 216.9, 134.8, 129.1, 128.3, 128.0, 45.6, 37.4, 37.2, 30.0 ppm.

*3-(Phenylselanyl)cyclohexanone* (**26**) [71]: Isolated as a yellow oil in 88% yield (0.071 g) without purification. ^1^H NMR (200 MHz, CDCl_3_) δ = 7.70–7.50 (m, 2 H, CH), 7.30–7.20 (m, 3 H, CH), 3.45 (tt, *J* = 4.4 and 10.5 Hz, 1 H, CH), 2.74 (ddt, *J* = 4.4, 13.7 and 1.5 Hz, 1 H, CH_2_), 2.40 (dd, *J* = 10.5 and 13.7 Hz, 1 H, CH_2_), 2.36–2.06 (m, 4 H, CH_2_), 1.87–1.51 (m, 2 H, CH_2_) ppm; ^13^C NMR (50.31 MHz, CDCl_3_) δ = 208.8, 135.6, 129.1, 128.1, 127.4, 48.6, 40.8, 40.1, 31.9, 25.1 ppm.

*3-(Phenylselanyl)cycloheptanone* (**27**) [48]: Isolated as a yellow oil in 95% yield (0.082 g) without purification. ^1^H NMR (200 MHz, CDCl_3_) δ = 7.70–7.50 (m, 2 H, CH), 7.40–7.30 (m, 3 H, CH), 3.50 (dddd, *J* = 2.8, 4.0, 9.6, and 9.8 Hz, 1 H, CH), 2.85 (ddd, *J* = 0.9, 4.0, and 14.9 Hz, 1 H, CH_2_), 2.75 (dd, *J* = 9.6 and 14.9 Hz, 1 H, CH_2_), 2.55–2.35 (m, 2 H, CH_2_), 2.15–2.05 (m, 1 H, CH_2_) 1.86–1.44 (m, 5 H, CH_2_) ppm; ^13^C NMR (50.31 MHz, CDCl_3_) δ = 211.8, 135.0, 129.1, 128.8, 128.0, 50.3, 43.9, 38.8, 37.7, 29.1, 23.7 ppm.

*tert-Butyl ((1R,2S/R-4-oxo-2-(phenylselanyl)cyclopentyl)carbamate* (**28**): Obtained as a mixture of diastereomers (75/25) from which the major one was isolated as a yellow oil in 62% yield (0.05 g) after flash column chromatography, eluent petroleum spirit/ethyl acetate (95:5). R*_f_* = 0.28 (c-Hex/EtOAc 5:1); ν_max_= 3370, 3327, 2976, 2930, 1747, 1721, 1675, 1512, 1473, 1435, 1391, 1366, 1156, 1018 cm^−1^. ^1^H NMR (400 MHz, CDCl_3_) δ = 7.70–7.50 (m, 2 H, CH), 7.40–7.20 (m, 3 H, CH), 4.77 (d, 1 H, *J* = 5.0 Hz, NH), 4.15–4.01 (m, 1 H, CH), 3.52 (q, 1 H, *J* = 8.0 Hz, CH), 2.81 (dd, 1 H, *J* = 7.6 and 15.1 Hz, CH_2_), 2.77 (dd, 1 H, *J* = 7.3 and 14.8 Hz, CH_2_), 2.3 (dd, 1 H, *J* = 9.1 and 19.5 Hz, CH_2_), 2.2 (dd, 1 H, *J* = 8.6 and 19.6 Hz, CH_2_), 1.4 (s, 9 H, CH_3_) ppm; ^13^C NMR (50.31 MHz, CDCl_3_) δ = 212.9, 155.1, 135.9, 129.1, 128.6, 126.4, 53.9, 45.2, 44.8, 42.2, 28.8 ppm. HRMS (ESI^+^) C_16_H_21_NO_3_NaSe (MNa^+^) calcd: 378.0584; found: 378.0585.

*Methyl-2-(1-hydroxy-4-oxo-6-(phenylselanyl)cyclohex-2-en-1-yl)acetate* (**29**): Isolated as a colorless oil in 29% yield (0.031g) after flash column chromatography, eluent cyclohexane/ethyl acetate (2:1). R*f* = 0.22 (c-Hex/EtOAc; 2:1). ν_max_ = 3454, 3056, 2985, 2955, 2925, 2852, 1732, 1685, 1619, 1577, 1521,1437, 1354, 1265 cm^-1^. ^1^H NMR (500 MHz, CDCl_3_), δ = 7.64–7.57 (m, 2 H, CH), 7.36–7.28 (m, 3 H, CH), 6.91 (d, 1 H, *J* = 10.2 Hz, CH), 6.02 (d, 1 H, *J* = 10.2 Hz, CH), 4.18 (s, 1 H, OH), 3.74 (dd, 1 H, *J* = 4.3 and 14.3 Hz, CH), 3.75 (s, 3 H, CH_3_), 3.30 (d, 1 H, *J* = 16.2 Hz, CH), 3.11 (dd, 1 H, *J* = 10.0 and 17.0 Hz, CH), 2.89 (dd, 1 H, *J* = 4.3 and 17.0 Hz, CH), 2.80 (d, 1 H, *J* = 16.2 Hz, CH) ppm; ^13^C NMR (125.77 MHz, CDCl_3_): 196.8, 171.9, 149.3, 135.2, 129.4, 129.1, 128.3, 128.2, 70.0, 52.22, (52.24), 50.7, 42.6, 42.5 ppm. HRMS (ESI^+^) C_15_H_16_O_4_NaSe (MNa^+^) calcd: 363.0111; found: 363.0098.

*3-((3-Phenylpropyl)selanyl)cyclohexanone* (**33**): Isolated as a yellow oil in 65% yield (0.044 g) and purified by flash column chromatography, eluent cyclohexane/ethyl acetate (9:1). R*_f_* = 0.28 (c-Hex/EtOAc 9:1); ν_max_ = 3026, 2928, 2858, 2362, 1714, 1602, 1495, 1454, 1274, 1122, 1030 cm^-1^. ^1^H NMR (400 MHz, CDCl_3_) δ = 7.28–7.25 (m, 2 H, CH), 7.19–7.15 (m, 3 H, CH), 3.18 (tt, 1 H, *J* = 3.9 and 10.5 Hz, CH), 2.76 (ddt, 1 H, *J* = 1.6, 4.5 and 14.2 Hz, CH_2_), 2.70 (t, *J* = 7.4 Hz, 2 H, CH_2_), 2.60 (t, 2 H, *J* = 7.4 Hz, CH_2_), 2.47 (ddd, 1 H, *J* = 1.1, 10.9, and 14.2 Hz, CH_2_), 2.39–2.26 (m, 2 H, CH_2_), 2.19–2.06 (m, 2 H, CH_2_), 1.97 (p, 2 H, *J* = 7.6 Hz, CH_2_), 1.83–1.64 (m, 2 H, CH_2_) ppm; ^13^C NMR (100 MHz, CDCl_3_) δ = 208.9, 141.3, 128.4, 128.3, 125.9, 49.2, 40.9, 35.9, 35.8, 32.5, 32.4, 25.4, 22.5 ppm. HRMS (ESI^+^) C_15_H_20_ONaSe (MNa^+^) calcd: 319.0577; found: 319.0568.

*(3S,4S/R)-3-((tert-Butyldimethylsilyl)oxy)-4-((3-phenylpropyl)selanyl)cyclopentanone* (**34**): Isolated as a 89/11 mixture of undetermined diastereomers from which the major one has been isolated as a yellow oil in 49% yield (0.050 g) by flash column chromatography, eluent cyclohexane/ethyl acetate (95:5). R*_f_* = 0.32 (c-Hex/EtOAc; 95:5); ν_max_ = 3085, 3062, 3026, 2953, 2927, 2855, 1748, 1604, 1496, 1455, 1253, 1102, 1066, 1026 cm^-1^. ^1^H NMR (400 MHz, CDCl_3_) δ = 7.29–7.26 (m, 2 H, CH), 7.20–7.15 (m, 3 H, CH), 4.41 (dt, 1 H, *J* = 2.8 and 5.4 Hz, CH), 3.45–3.39 (m, 1 H, CH), 2.92–2.85 (m, 1 H, CH_2_), 2.76–2.52 (m, 5 H, CH_2_), 2.23–2.11 (m, 2 H, CH_2_), 2.09–1.93 (m, 2 H, CH_2_), 0.85 (s, 9 H, CH_3_), 0.06 (s, 3 H, CH_3_), 0.04 (s, 3 H, CH_3_) ppm; ^13^C NMR (100 MHz, CDCl_3_) δ = 215.0, 128.4, 128.3, 126.0, 125.91, 75.2, 46.1, 43.1, 41.0, 35.8, 31.9, 25.6, 23.7, −4.7, −4.8 ppm. HRMS (ESI^+^) C_20_H_32_O_2_NaSeSi (MNa^+^) calcd: 435.1234; found: 435.1235 [α]D = −17 (c = 0.5, CHCl_3_).

*(2R)-Methyl 2-((tert-butoxycarbonyl)amino)-3-((3-oxobutyl)selanyl)propanoate* (**35**): Isolated as a yellow oil in 30% yield (0.021 g) after flash column chromatography, eluent cyclohexane/ethyl acetate (3:1). Rf = 0.17 (c-Hex/EtOAc; 3:1); ν_max_ = 3379, 3344, 2977, 2953, 2929, 1708, 1436, 1365, 1249, 1212, 1160, 1050, 1007 cm^-1^. ^1^H NMR (600 MHz, CDCl_3_) δ = 5.35 (d, 1 H, *J* = 6.5 Hz, NH), 4.59 (d, 1 H, *J* = 6.9 Hz, CH), 3.74 (s, 3 H, CH_3_), 3.05–2.98 (m, 2 H, CH_2_), 2.85–2.79 (m, 2 H, CH_2_), 2.74–2.70 (m, 2 H, CH_2_), 2.14 (s, 3 H, CH_3_), 1.43 (s, 9 H, CH_3_) ppm; ^13^C-NMR (125.77 MHz, CDCl_3_) δ = 206.7, 171.5, 154.9, 80.0, 53.5, 52.6, 44.4, 29.9, 28.3, 26.6, 17.5 ppm. HRMS (ESI^+^) C_13_H_23_NO_5_NaSe (MNa^+^) calcd: 376.0639; found: 376.0653 [α]D = +19 (c = 0.5, CHCl_3_).

*Methyl 2-((tert-butoxycarbonyl)amino)-3-((3-oxocyclohexyl)selanyl)propanoate* (**36**): Isolated as a yellow oil in 53% yield (0.050 g) as mixture of two inseparable diastereomers (66/34) after flash column chromatography, eluent cyclohexane/ethyl acetate (3:1). R*_f_* = 0.23 (c-Hex/EtOAc; 3:1); ν_max_ = 3358, 2976, 1742, 1704, 1500, 1436, 1392, 1365, 1346, 1248, 1215, 1048, 1007 cm^−1^. For ^1^H NMR (500 MHz, CDCl_3_) and ^13^C NMR (125.77 MHz, CDCl_3_) of the mixture, see Supplementary Materials. HRMS (ESI^+^) C_15_H_25_NO_5_NaSe (MNa^+^) calcd: 402.0796; found: 402.0779.

*Methyl-2-((tert-butoxycarbonyl)amino)-3-(((2S)-2-((tert-butoxycarbonyl)amino)-4-oxocyclopentyl)selanyl)propanoate* (**37**): Isolated as a white solid, mixture of diastereomers (55/45) in a 30% yield (0.046 g) after recrystallization from a concentrated DCM solution which was layered with c-Hex. R*_f_* = 0.28 (c-Hex/EtOAc; 5:1). m.p. 114–116 °C. ν_max_ = 3362, 2932, 1743, 1677, 1511, 1437, 1366, 1317, 1278, 1253, 1228, 1161, 1045, 1017 cm^−1^. For ^1^H NMR (600 MHz, CDCl_3_) and ^13^C NMR (150.9 MHz, CDCl_3_) of the mixture, see Supplementary Materials. HRMS (ESI^+^) C_19_H_32_N_2_O_7_NaSe (MNa^+^) calcd: 503.1272; found: 503.1271.

## 4. Conclusions

An efficient, fast, and safe protocol for seleno-Michael-type addition reactions has been reported. The method represents a good way to achieve the in situ reduction of diphenyl diselenide **1** using a biphasic Zn-EtOAc-HCl(aq) mixture. The resultant nucleophilic selenenylating mixture was shown to react with a range of Michael acceptors in order to provide the corresponding Se-adducts. Yields are often high and these were found to depend on the electrophilicity of the substrate. Alternative diselenides **32** and **33** were successfully used, affording Se-alkylated versions of protected selenocysteine.

## Figures and Tables

**Table 1:** Telescoped diselenide reduction-conjugate addition to α,β-unsaturated carbonyl derivatives **2**–**10**.

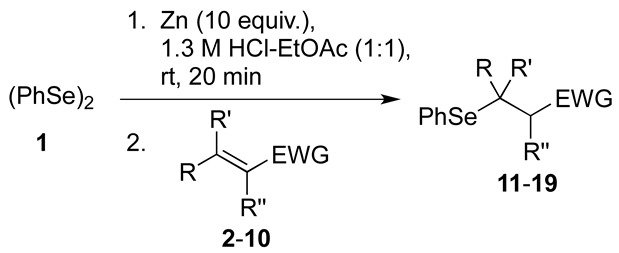			
Entry	Substrate ^a^	Se-Adduct	Yield ^b^
1	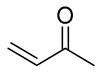 **2**	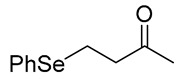 **11**	91%
2	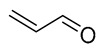 **3**	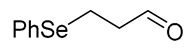 **12**	44%
3	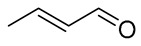 **4**	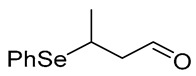 **13**	70%
4	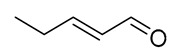 **5**	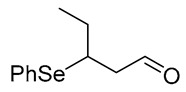 **14**	60%
5	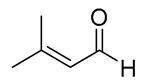 **6**	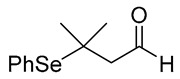 **15**	22%
6	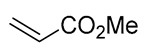 **7**	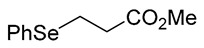 **16**	79%
7	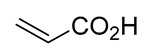 **8**	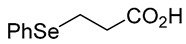 **17**	60%
8	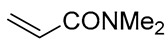 **9**	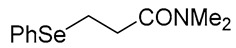 **18**	25%
9	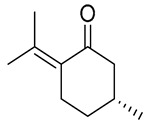 **10**	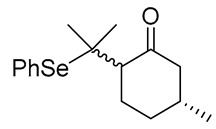 **19**	95% ^c^ (62:38)

^a^ Phenyl vinyl sulfone, acrylonitrile, and chalcones did not afford the corresponding target compound. ^b^ Quoted for analytically pure material, obtained either directly, or following purification by flash column chromatography. ^c^ Obtained as an undetermined mixture of diastereomers (62:38) determined by the ^1^H-NMR of the crude integrating the doublets of the methyl group at 1.02 ppm for the major isomer and 0.97 for the minor.

**Table 2:** Telescoped diselenide reduction-conjugate addition to cyclic enones **20**–**24**.

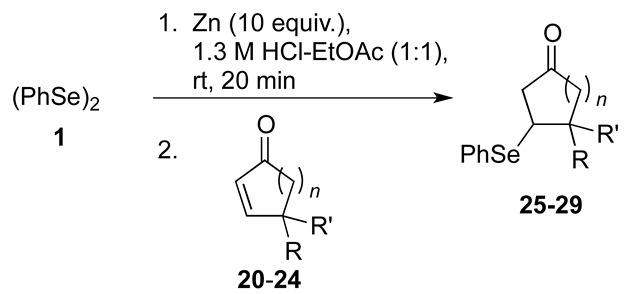			
Entry	Substrate	Se-Adduct	Yield ^a^
1	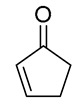 **20**	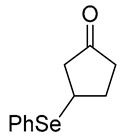 **25**	93%
2	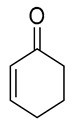 **21**	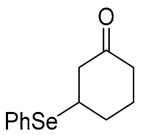 **26**	88%
3	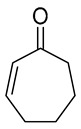 **22**	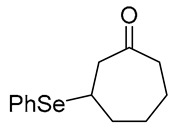 **27**	95%
4	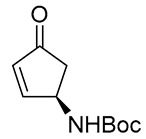 **23**	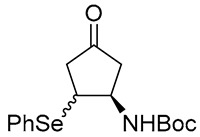 **28**	90% ^b^ (75:25)
5	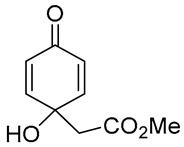 **24**	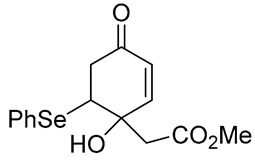 **29**	29% ^c^

^a^ Quoted for analytically pure material, obtained either directly or following purification by flash column chromatography. ^b^ NMR conversion of the diastereomeric mixture from which the major isomer was isolated in 62% yield after flash chromatography. Even if the attempts to clarify the relative configuration by NOE experiments failed and we were not able to obtain crystals suitable for X-ray analysis, in accordance with similar sulphur derivatives reported by some of us, we can assume a *trans*-geometry between substituents in C-3 and C-4 [65,66]. ^c^ Obtained as a single undetermined diastereomer.

**Table 3:** Reaction of 1,2-bis(3-phenylpropyl)diselenide **30** and protected selenocystine **31** with enones.

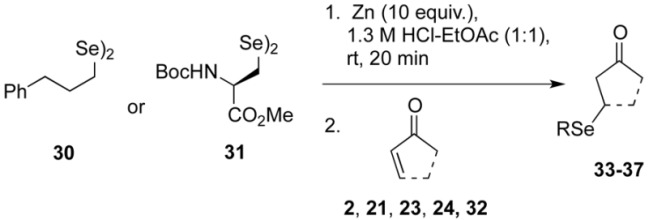			
Entry	Substrate	Se-Adduct	Yield ^a^
1	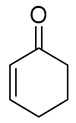 **21**	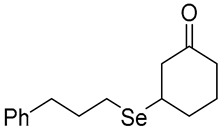 **33**	65%
2	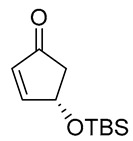 **32**	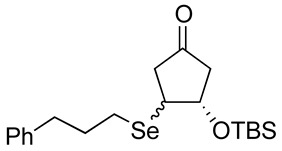 **34**	90% ^b^ (89:11)
3	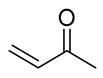 **2**	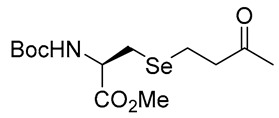 **35**	30%
4	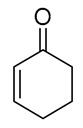 **21**	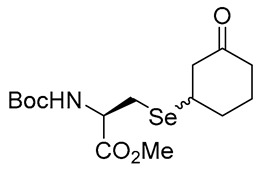 **36**	53% ^c^ (66:34)
5	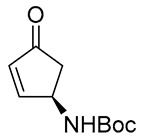 **23**	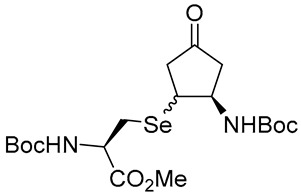 **37**	30% ^c^ (55:45)

^a^ Quoted for analytically pure material, obtained either directly or following purification by flash column chromatography. ^b^ NMR conversion of the diastereomeric mixture from which the major isomer was isolated in 49% yield after flash chromatography. In analogy with **28**, we can assume a *trans*-geometry between substituents in C-3 and C-4 [65,66]. ^c^ Obtained as an inseparable and undetermined mixture of diastereomers determined by the ^1^H-NMR of the crude.

## Supplementary Materials

The following are available online at https://www.mdpi.com/1420-3049/25/9/2018/s1, Figure of NMR spectra of all the synthesized compounds.
