# Supplementary material for: A Simple Zinc-Mediated Method for Selenium Addition to Michael Acceptors
Source: Molecules. 2020 Apr 26;25(9):2018. doi: 10.3390/molecules25092018 (PMC7249194; doi:10.3390/molecules25092018)
Supplement: Supplementary file 1 [file molecules-25-02018-s001.pdf]

Supporting Information

A Simple Zinc-Mediated Method for Selenium Addition to Electron-Poor Alkenes

Francesca Giulia Nacca, Bonifacio Monti, Eder João Lenardão, Paul Evans and Claudio Santi*

NMR spectra of compounds **11-19**; **25-29** and **33-37** **S-2**

NMR spectra

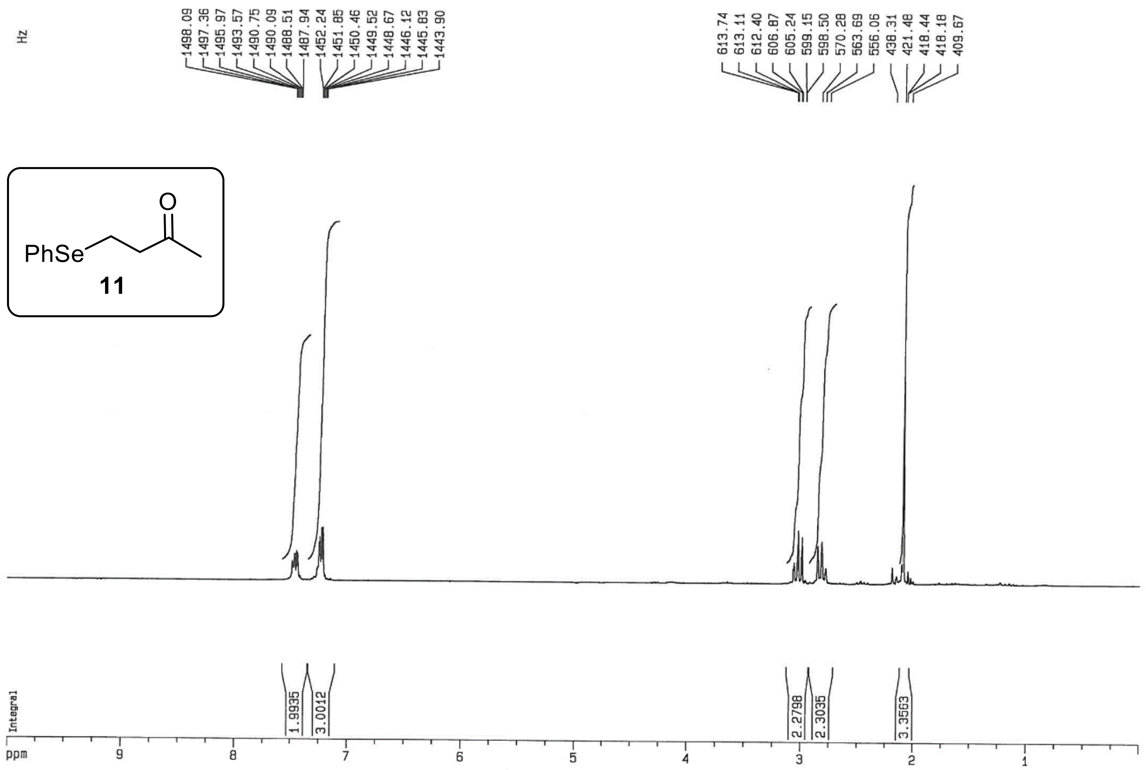
PhSe
O
11
Hz
1498.09
1497.36
1495.97
1493.57
1490.75
1490.09
1488.51
1487.94
1452.24
1451.85
1450.46
1449.52
1448.67
1446.12
1445.83
1443.90
613.74
613.11
612.40
606.87
605.24
599.15
598.50
570.28
563.69
556.06
438.31
421.48
418.44
418.18
409.67
Integral
1.9935
3.0012
2.2798
2.3035
3.3563
ppm
9
8
7
6
5
4
3
2
1

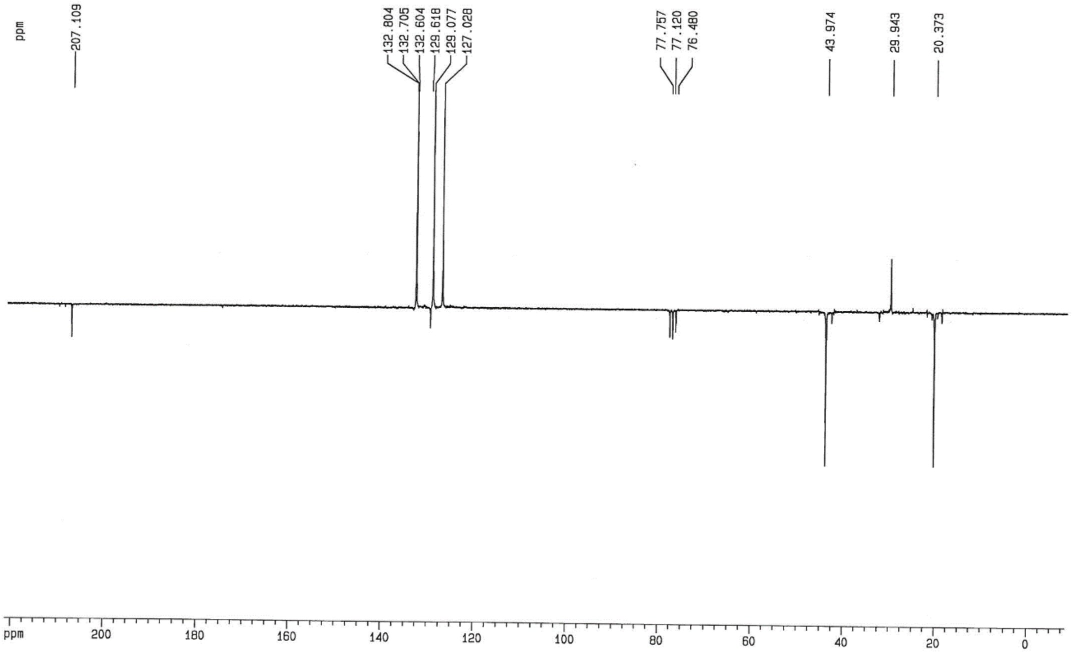
ppm
207.109
132.804
132.705
132.604
129.618
129.077
127.028
77.757
77.120
76.480
43.974
29.943
20.373
ppm
200
180
160
140
120
100
80
60
40
20
0

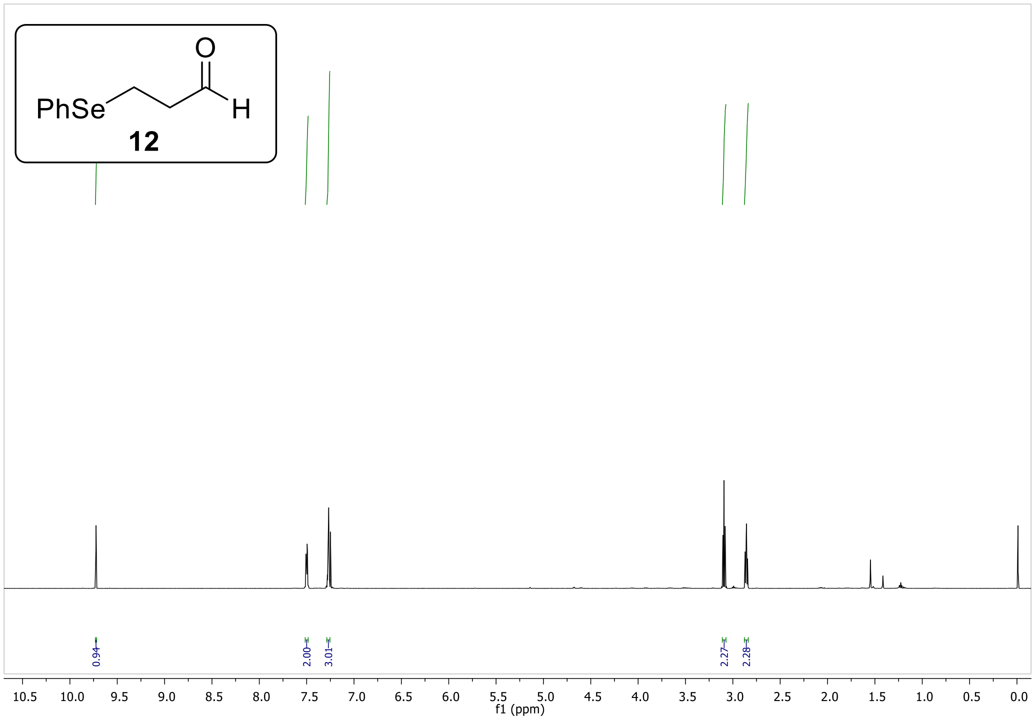
PhSe
O
H
12
0.94
2.00
3.01
2.27
2.28
10.5 10.0 9.5 9.0 8.5 8.0 7.5 7.0 6.5 6.0 5.5 5.0 4.5 4.0 3.5 3.0 2.5 2.0 1.5 1.0 0.5 0.0
f1 (ppm)

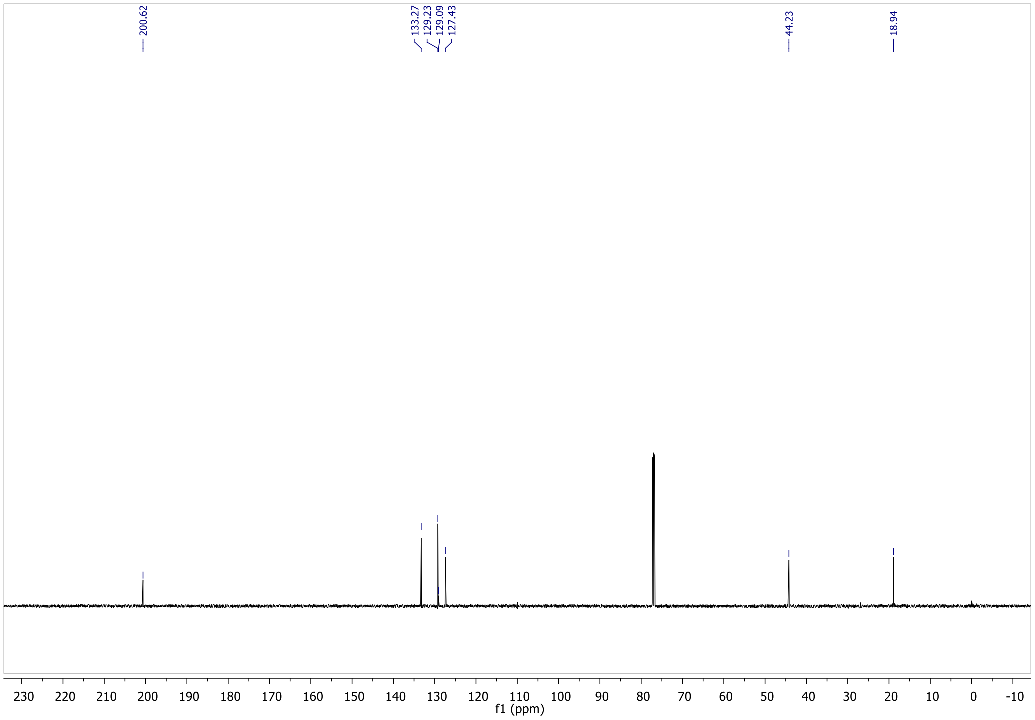
200.62
133.27
129.23
129.09
127.43
44.23
18.94
230 220 210 200 190 180 170 160 150 140 130 120 110 100 90 80 70 60 50 40 30 20 10 0 -10
f1 (ppm)

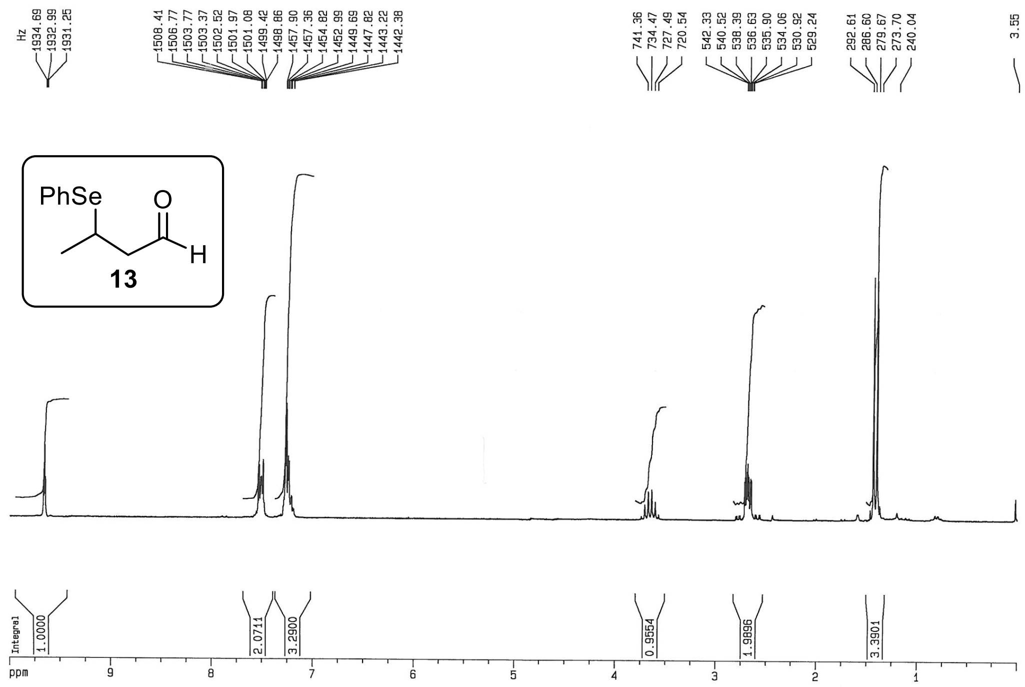
PhSe
O
H
13
Integral
1.0000
2.0711
3.2900
0.9554
1.9896
3.3901
ppm

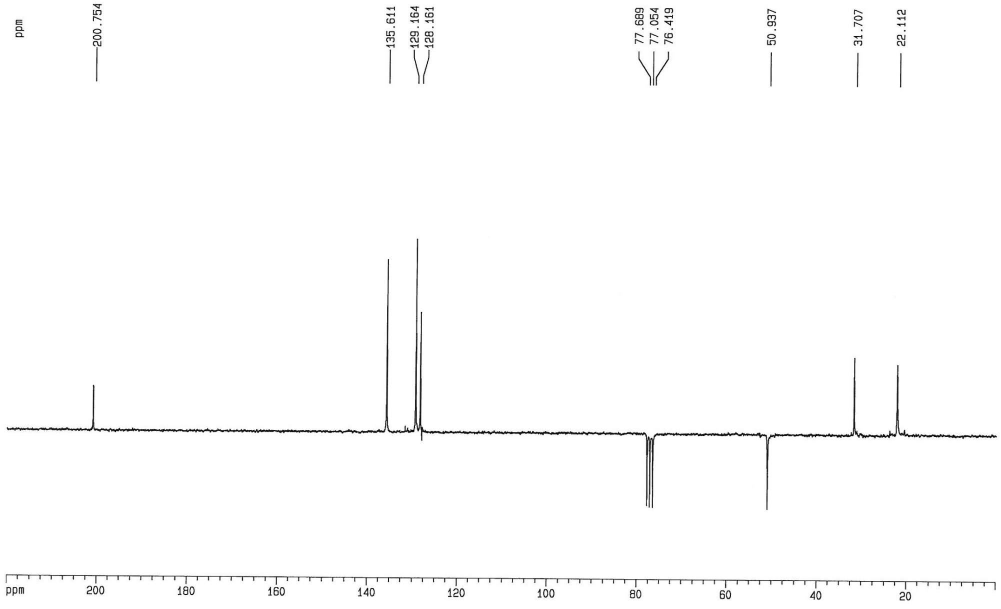
ppm
200.754
135.611
129.164
128.161
77.689
77.054
76.419
50.937
31.707
22.112

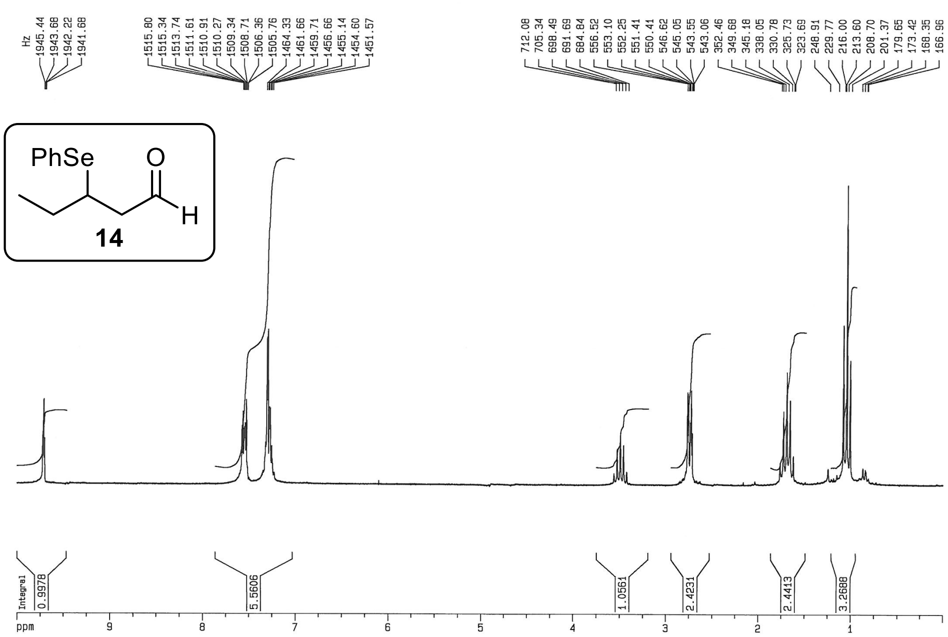
Hz
1945.44 1943.68 1942.22 1941.68
1515.80 1515.34 1513.74 1511.61 1510.91 1510.27 1509.34 1508.71 1506.36 1505.76 1464.33 1461.66 1459.71 1456.66 1455.14 1454.60 1451.57
712.08 705.34 698.49 691.69 684.84 556.52 553.10 552.25 551.41 550.41 546.62 545.05 543.55 543.06 352.46 349.68 345.18 338.05 330.78 325.73 323.69 248.91 229.77 216.00 213.60 208.70 201.37 179.65 173.42 168.35 166.96
PhSe
O
H
14
Integral
0.9978
5.5606
1.0561
2.4231
2.4413
3.2688
ppm 9 8 7 6 5 4 3 2 1

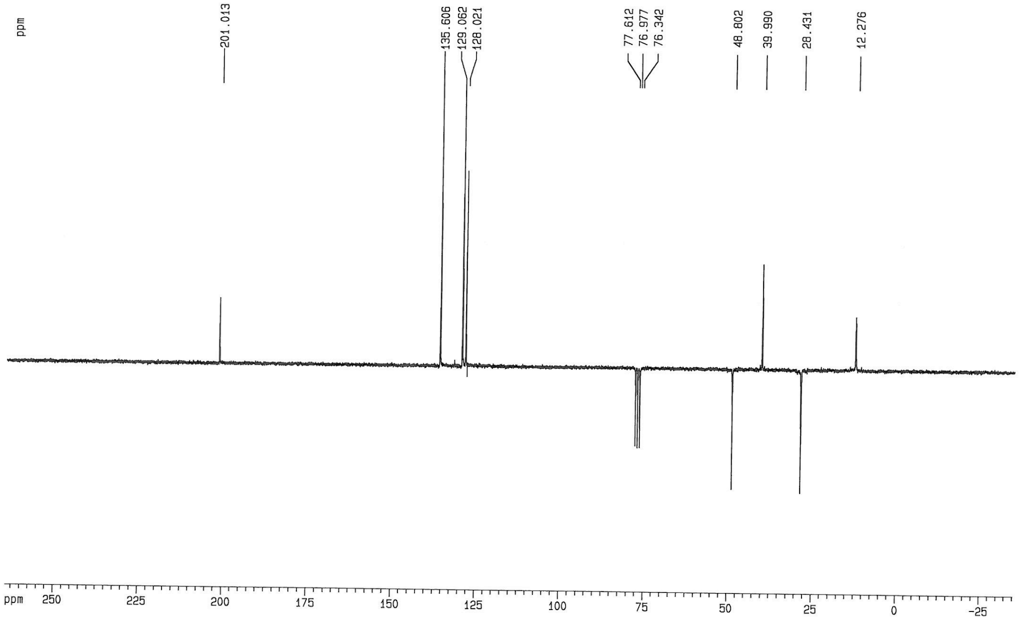
ppm
201.013
135.606
129.062
128.021
77.612
76.977
76.342
48.802
39.990
28.431
12.276
ppm 250 225 200 175 150 125 100 75 50 25 0 -25

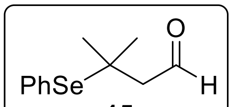

15

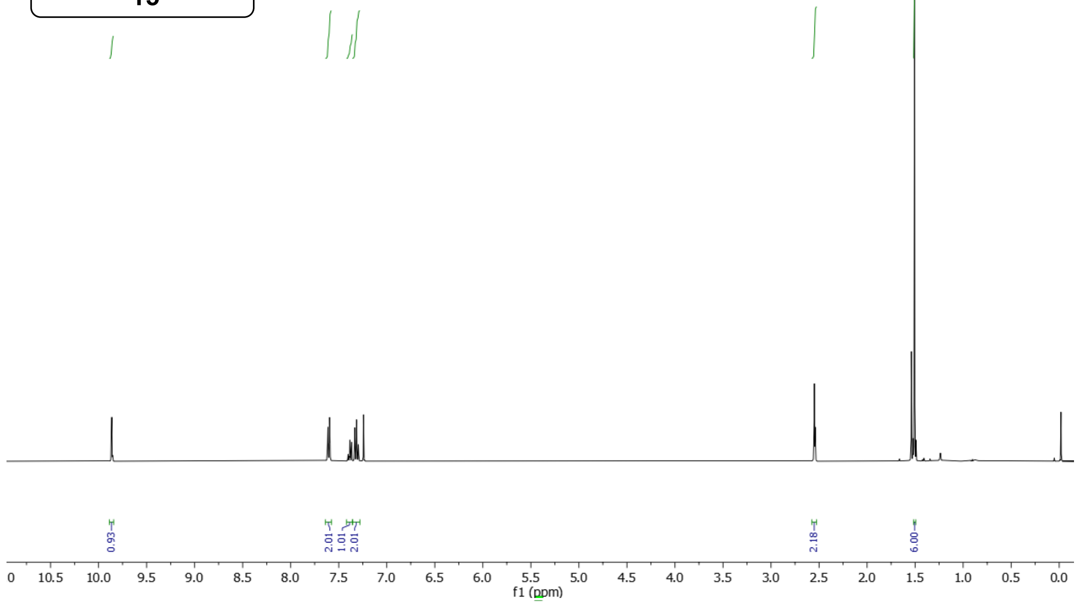
0.93
2.01
1.01
2.01
2.18
6.00
f1 (ppm)

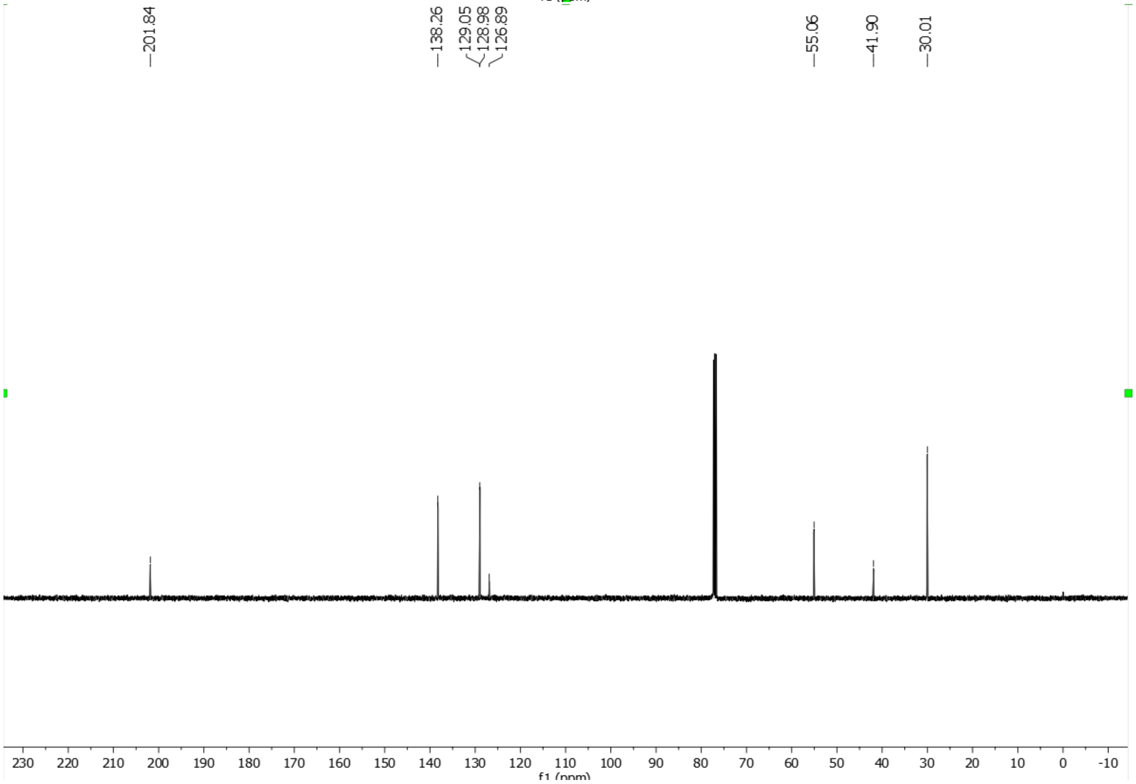
201.84
138.26
129.05
128.98
126.89
55.06
41.90
30.01
f1 (ppm)

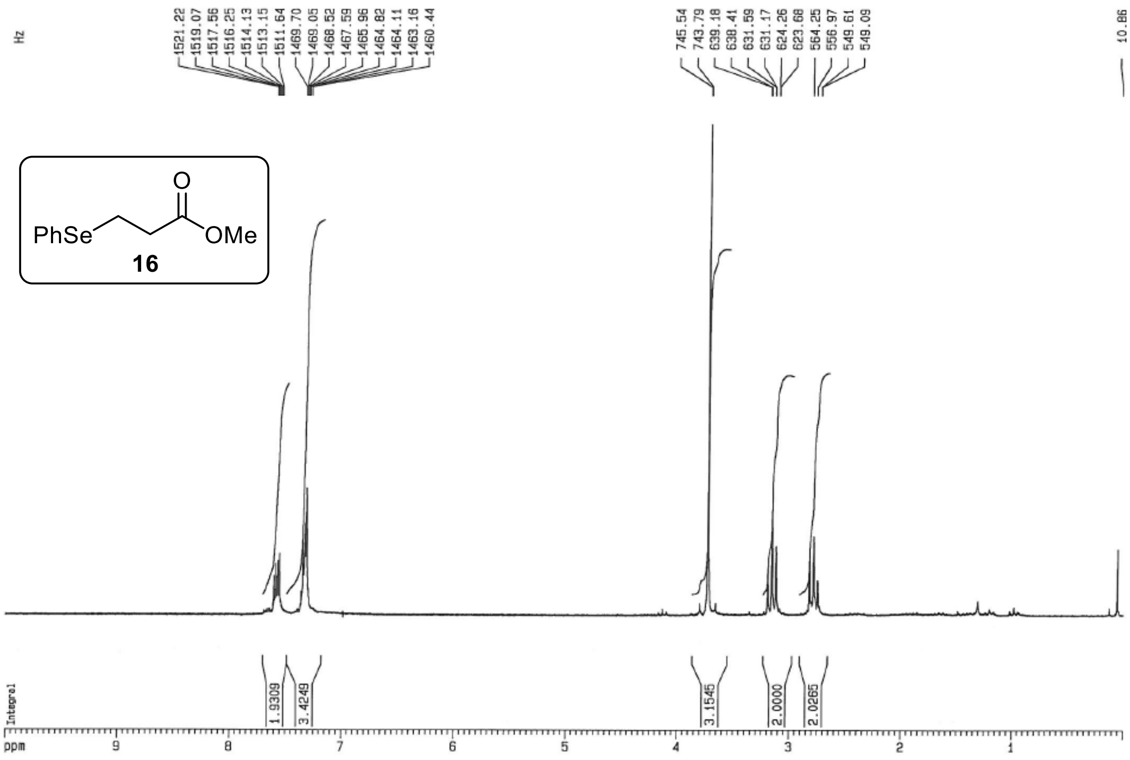
Hz
1521.22
1519.07
1517.56
1516.25
1514.13
1513.15
1511.64
1469.70
1469.05
1468.52
1467.59
1465.96
1464.82
1464.11
1463.16
1460.44
745.54
743.79
639.18
638.41
631.59
631.17
624.26
623.68
564.25
556.97
549.61
549.09
10.86
O
PhSe
OMe
16
Integral
1.9309
3.4249
3.1545
2.0000
2.0265
ppm
9
8
7
6
5
4
3
2
1

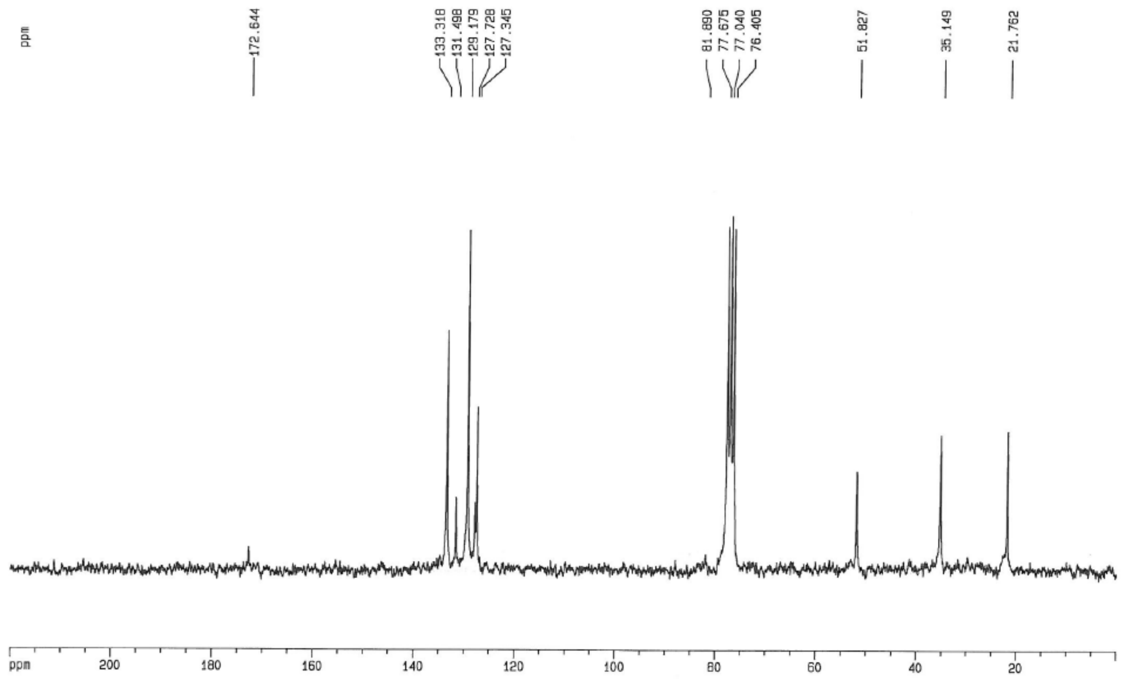
ppm
172.644
133.318
131.498
129.179
127.728
127.345
81.890
77.675
77.040
76.405
51.827
35.149
21.762
ppm
200
180
160
140
120
100
80
60
40
20

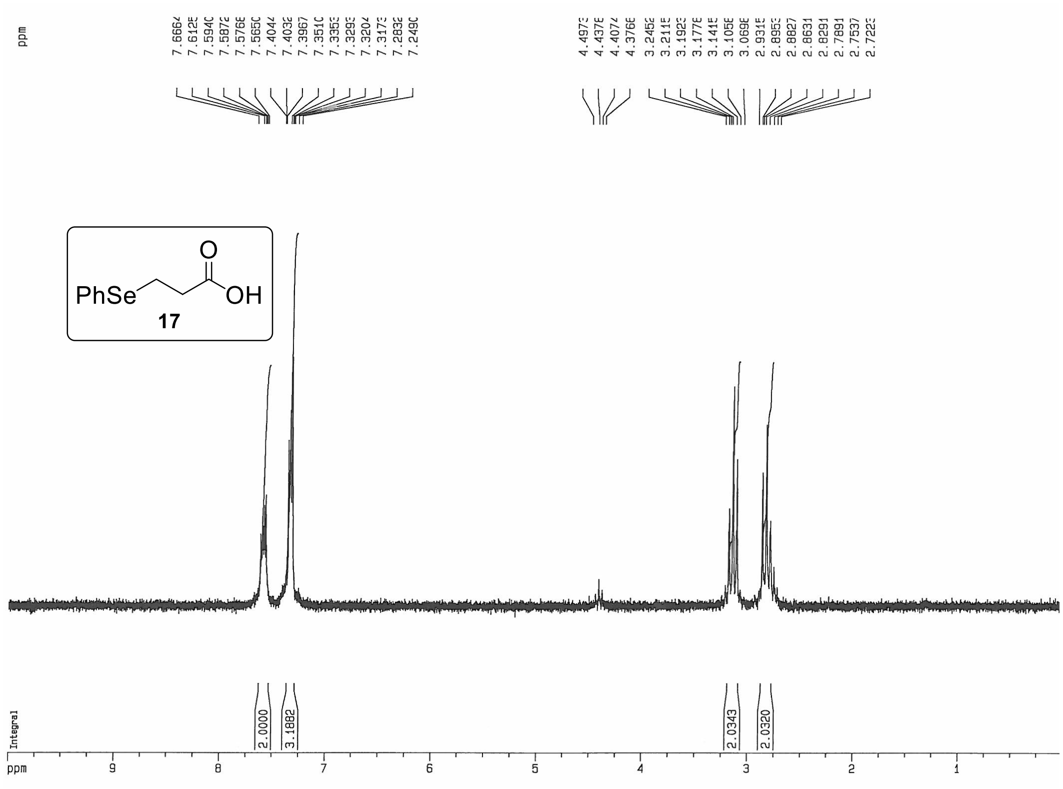

ppm
PhSe
O
OH
17
Integral
2.0000
3.1882
2.0343
2.0320
ppm

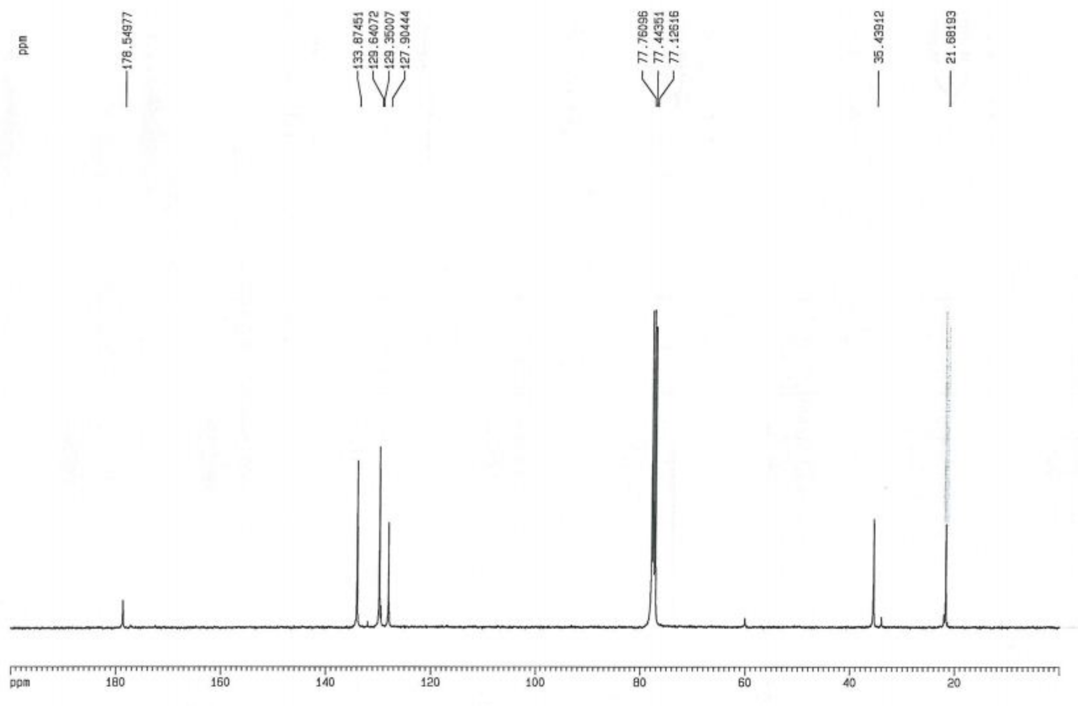

ppm
178.54977
133.87451
129.64072
129.35007
127.90444
77.76096
77.44351
77.12616
35.43912
21.68193
ppm

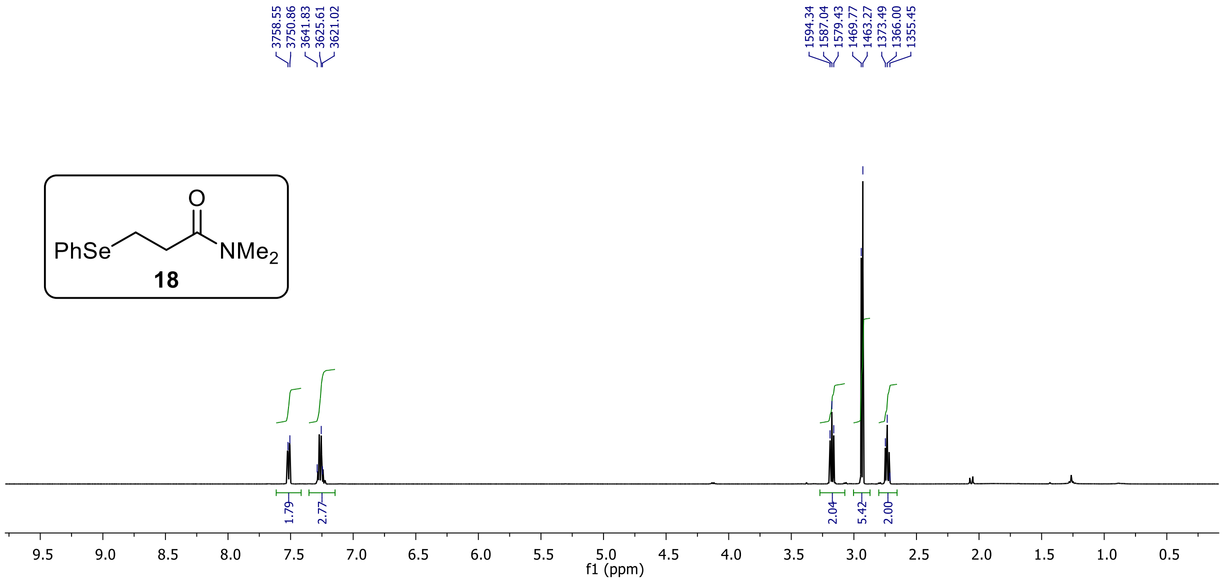
3758.55
3750.86
3641.83
3625.61
3621.02
1594.34
1587.04
1579.43
1469.77
1463.27
1373.49
1366.00
1355.45
PhSe
O
NMe2
18
1.79
2.77
2.04
5.42
2.00
9.5
9.0
8.5
8.0
7.5
7.0
6.5
6.0
5.5
5.0
4.5
4.0
3.5
3.0
2.5
2.0
1.5
1.0
0.5
f1 (ppm)

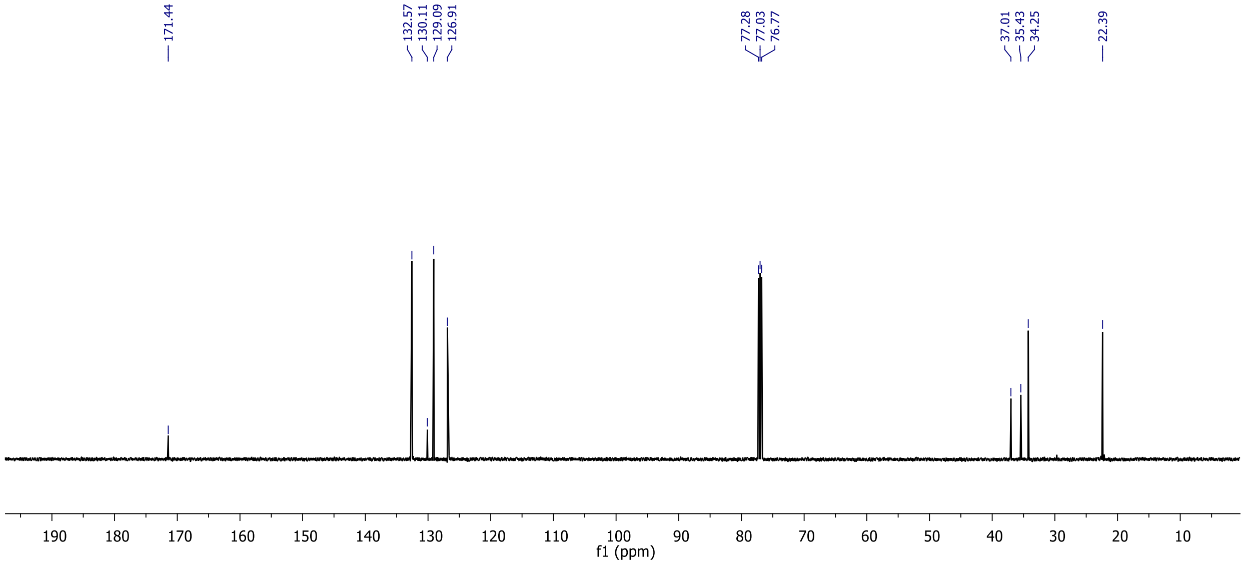
171.44
132.57
130.11
129.09
126.91
77.28
77.03
76.77
37.01
35.43
34.25
22.39
190
180
170
160
150
140
130
120
110
100
90
80
70
60
50
40
30
20
10
f1 (ppm)

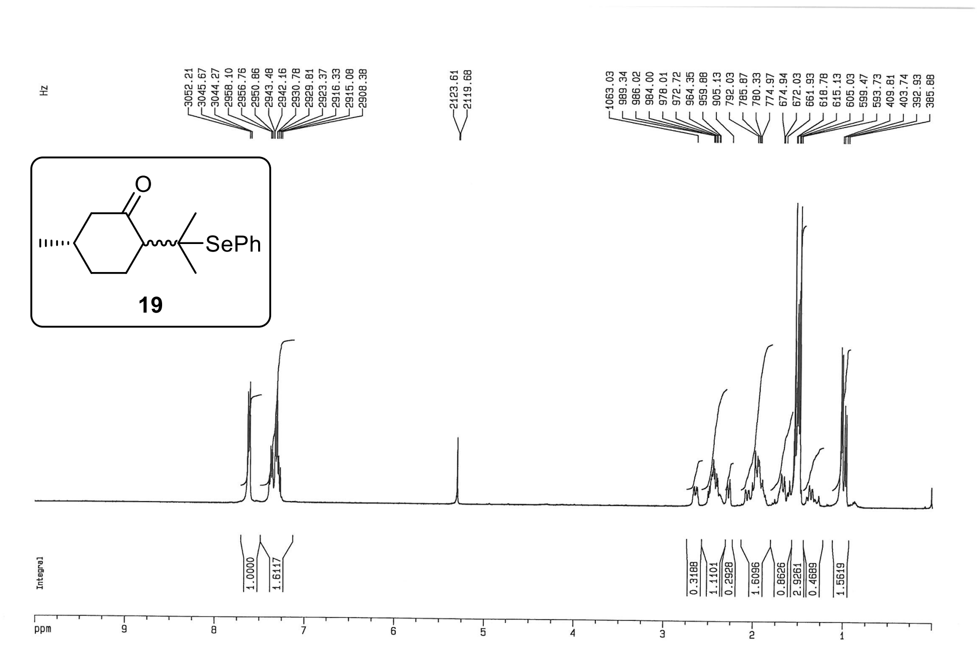
O
SePh
19
Hz
Integral
ppm

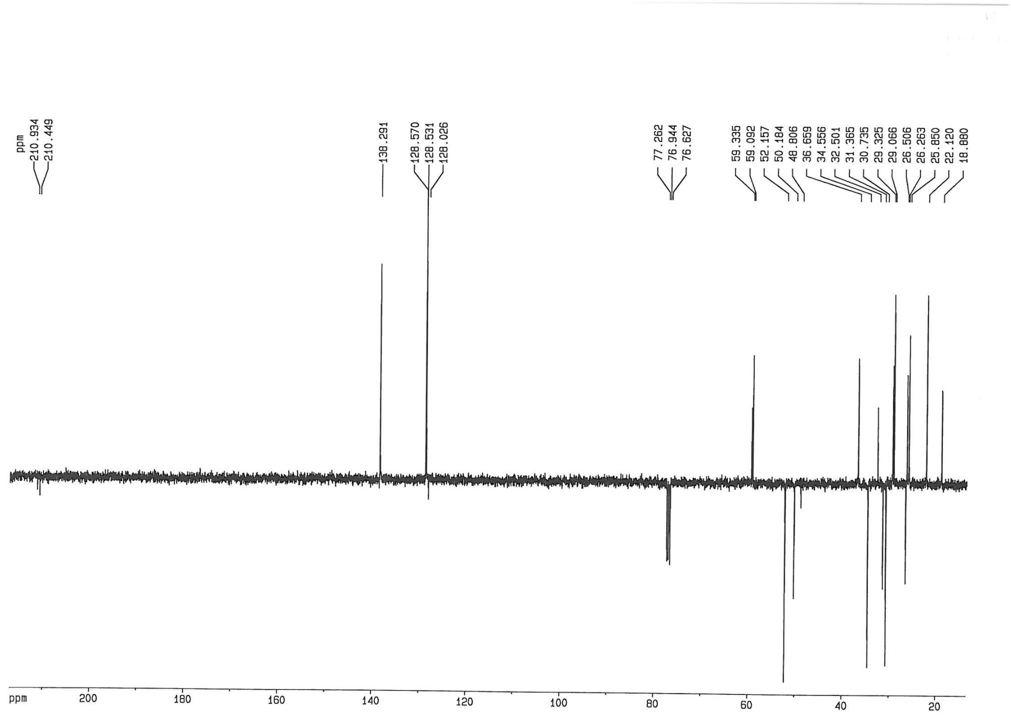
ppm

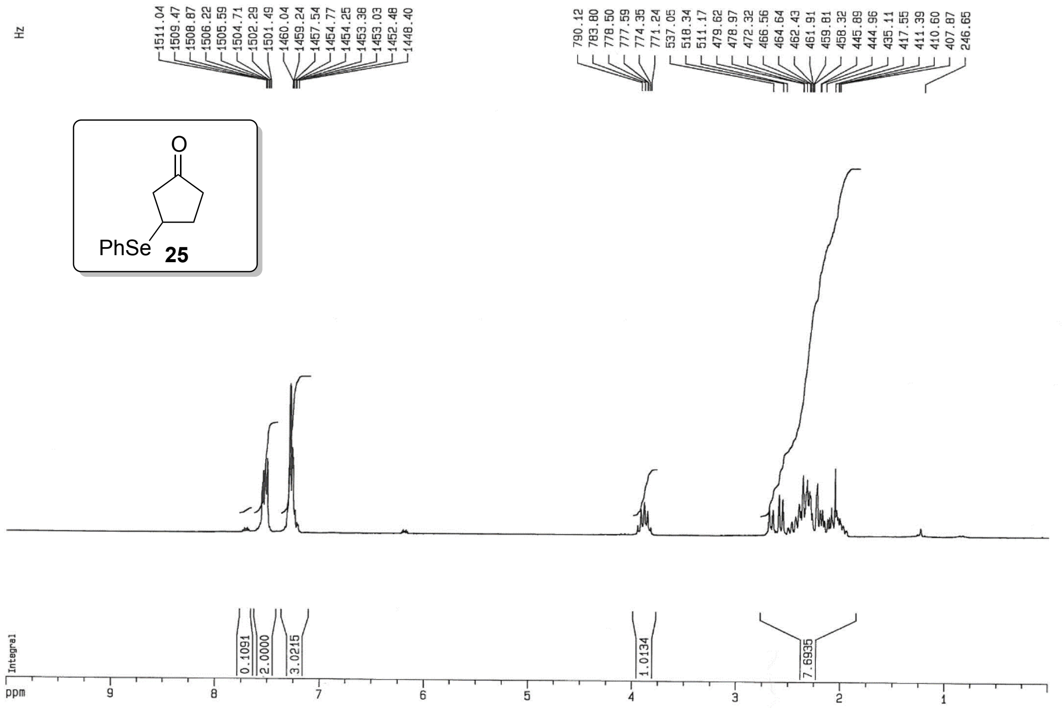
PhSe
25

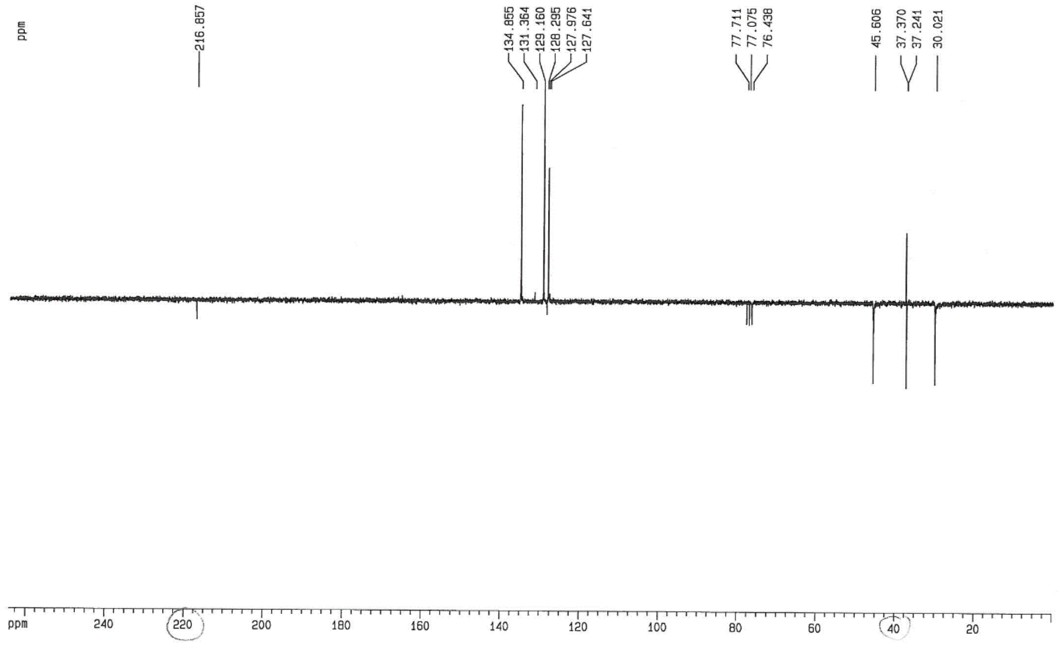

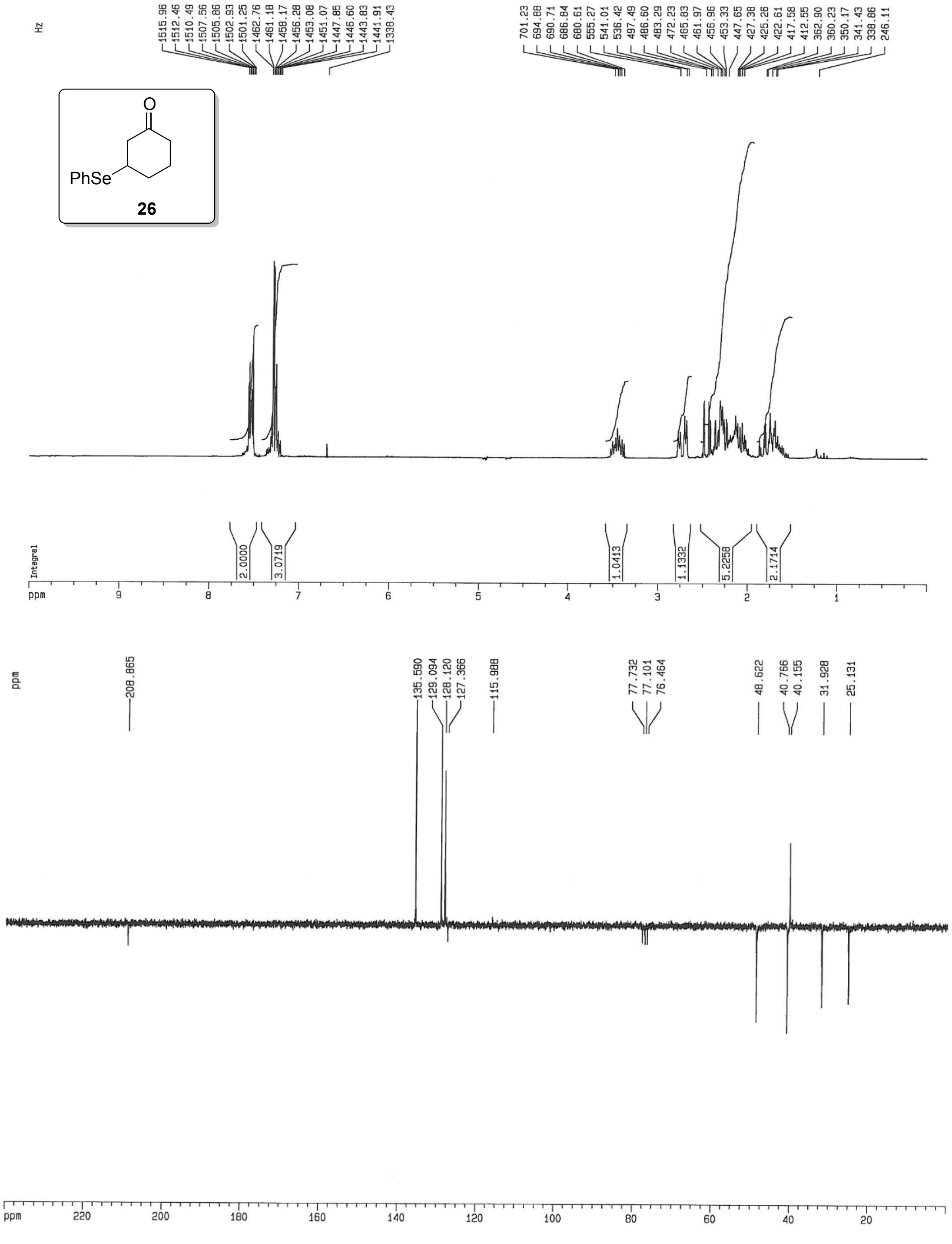
PhSe
26
Hz
1515.96
1512.46
1510.49
1507.56
1505.86
1502.93
1501.25
1462.76
1461.10
1458.17
1456.28
1453.08
1451.07
1447.85
1446.60
1443.83
1441.91
1338.43
701.23
694.88
690.71
686.84
680.61
555.27
541.01
536.42
497.49
486.60
483.29
472.23
465.83
461.97
456.96
453.33
447.65
427.38
425.26
422.61
417.58
412.55
362.90
360.23
350.17
341.43
338.86
246.11
Integral
2.0000
3.0719
1.0413
1.1332
5.2258
2.1714
ppm
208.865
135.590
129.094
128.120
127.366
115.988
77.732
77.101
76.464
48.622
40.766
40.155
31.928
25.131

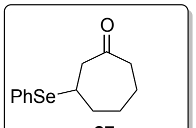
O
PhSe

27

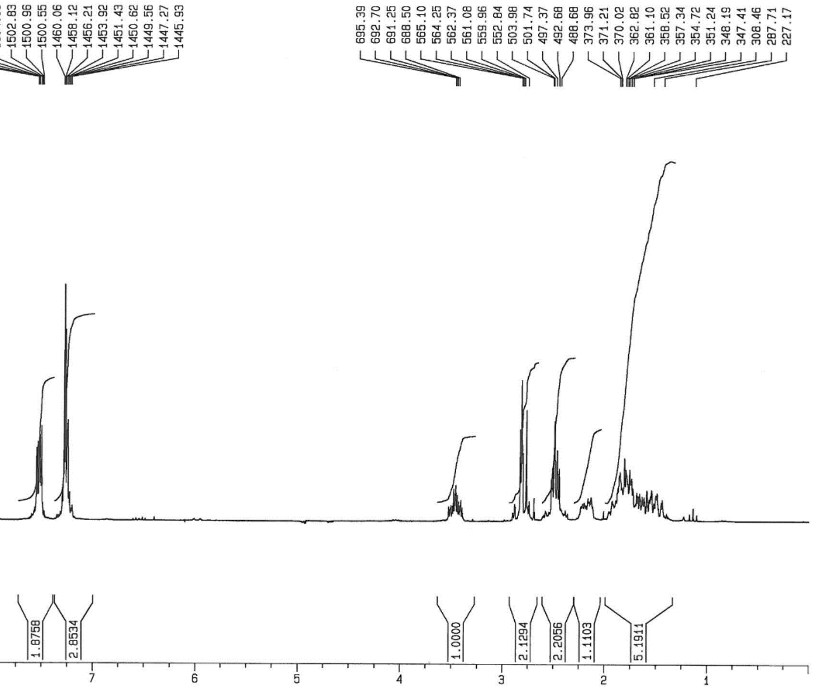
Hz
1510.55
1510.12
1508.34
1507.84
1504.63
1502.83
1500.96
1500.55
1460.06
1458.12
1456.21
1453.92
1451.43
1450.62
1449.56
1447.27
1445.93
695.39
692.70
691.25
688.50
565.10
564.25
562.37
561.08
559.96
552.84
503.98
501.74
497.37
492.68
488.68
373.96
371.21
370.02
362.82
361.10
358.52
357.34
354.72
351.24
348.19
347.41
308.46
287.71
227.17
Integral
1.8758
2.8534
1.0000
2.1294
2.2056
1.1103
5.1911
ppm
9
8
7
6
5
4
3
2
1

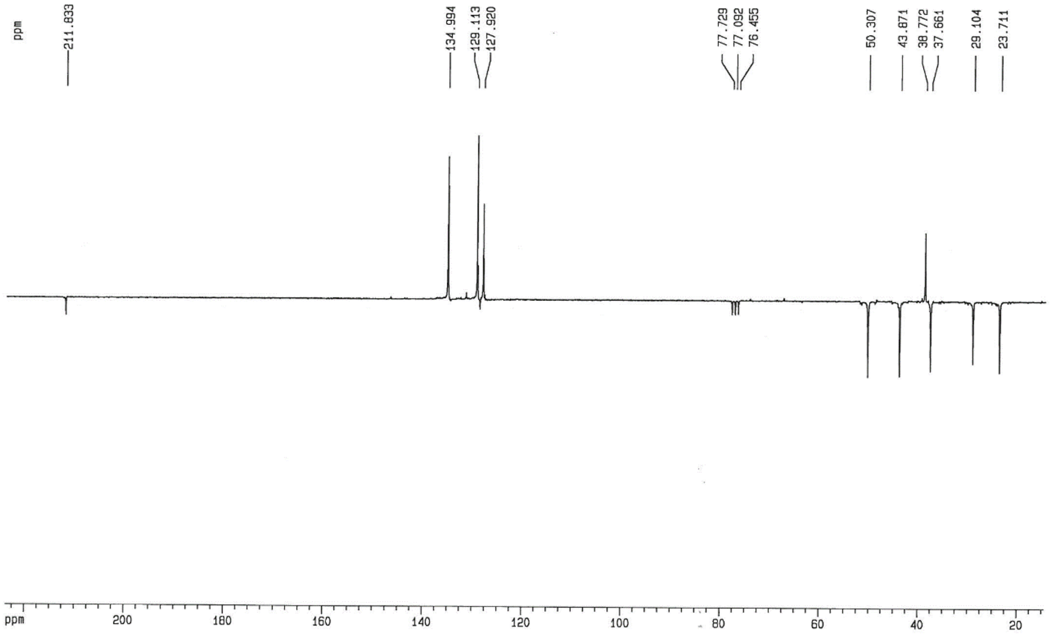
ppm
211.833
134.994
129.113
127.920
77.729
77.092
76.455
50.307
43.871
38.772
37.661
29.104
23.711
ppm
200
180
160
140
120
100
80
60
40
20

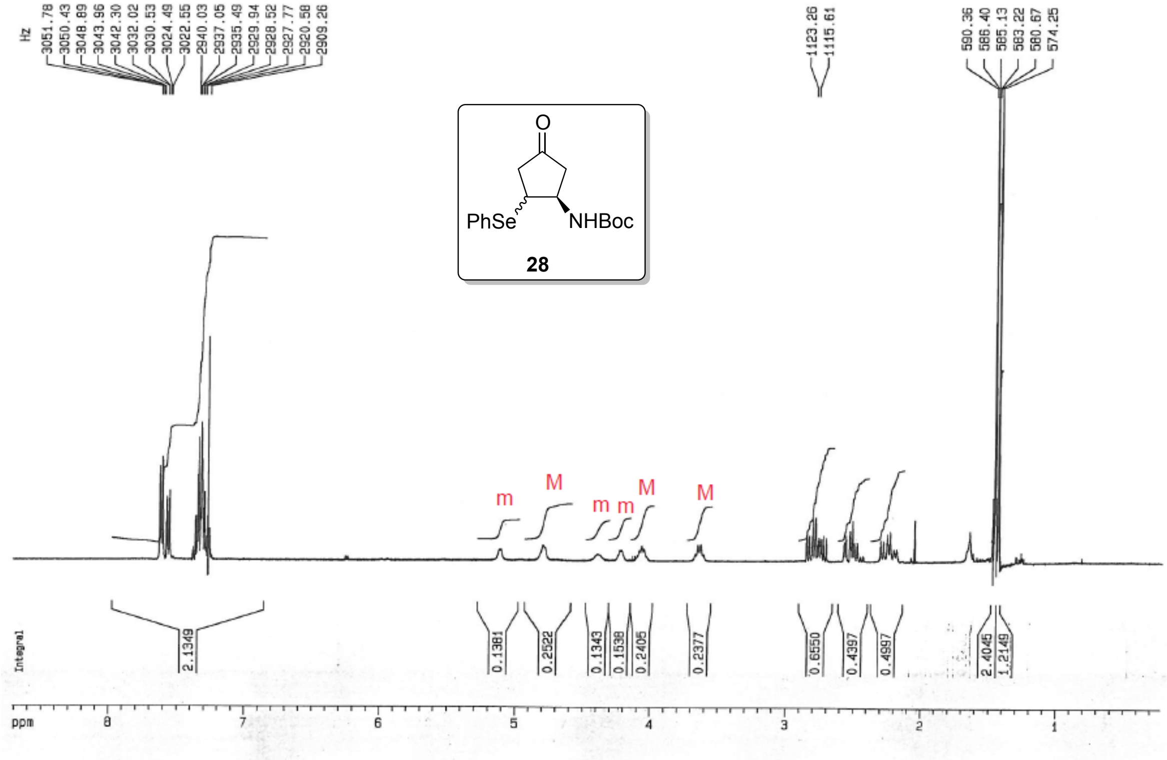

Hz
3051.78 3050.43 3048.89 3043.96 3042.30 3032.02 3030.53 3024.49 3022.55 2940.03 2937.05 2935.49 2929.94 2928.52 2927.77 2920.58 2909.26
1123.26 1115.61
590.36 586.40 585.13 583.22 580.67 574.25
O
PhSe
NHBoc
28
m
M
m
m
M
M
Integral
2.1349
0.1381
0.2522
0.1343
0.1538
0.2405
0.2377
0.6550
0.4397
0.4997
2.4045
1.2149
ppm
8
7
6
5
4
3
2
1

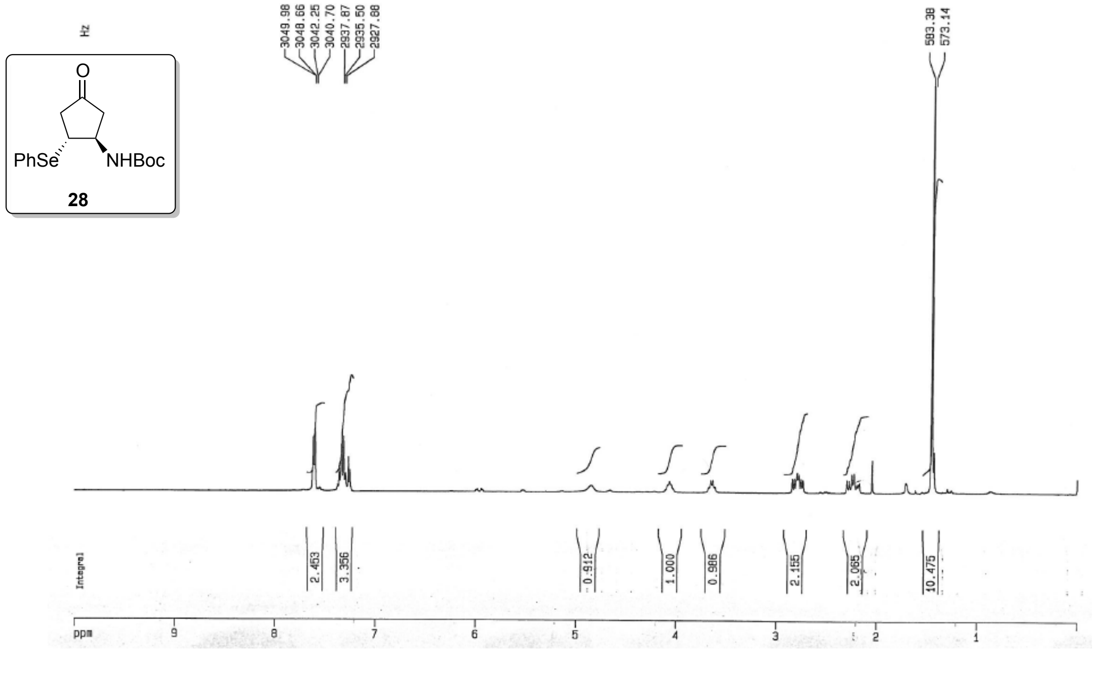

Hz
3049.98 3048.66 3042.25 3040.70 2937.87 2935.50 2927.88
583.38 573.14
O
PhSe
NHBoc
28
Integral
2.453
3.356
0.912
1.000
0.986
2.155
2.065
10.475
ppm
9
8
7
6
5
4
3
2
1

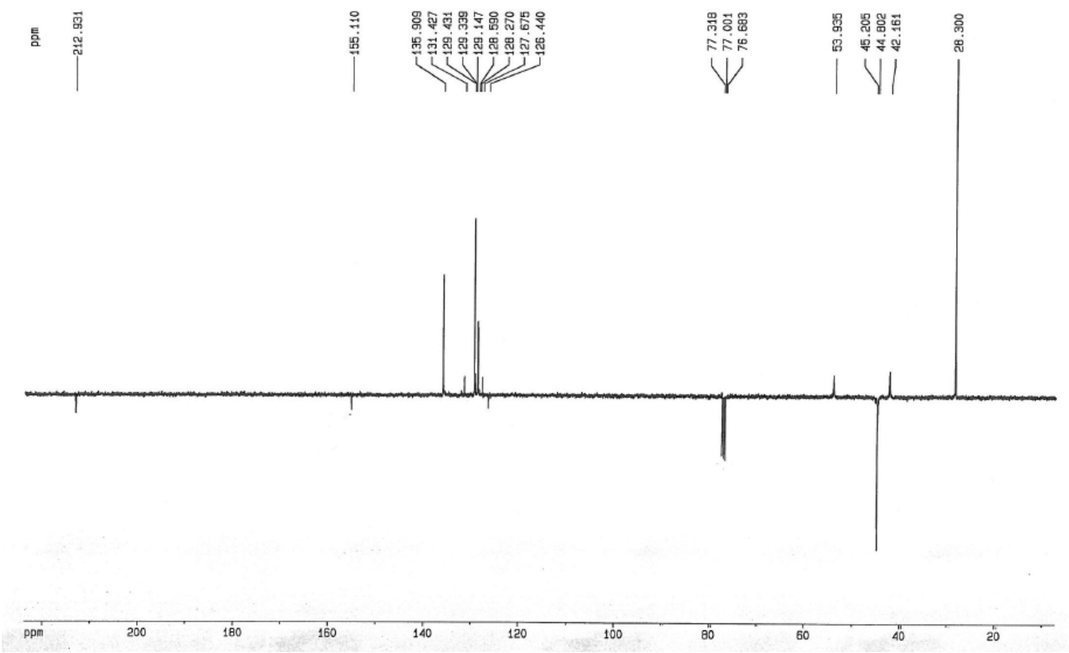

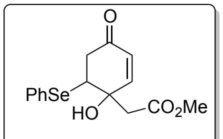

29

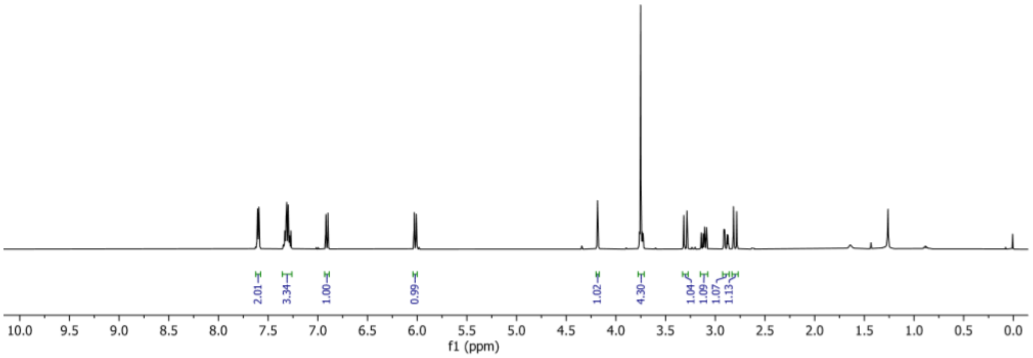

2.01
3.34
1.00
0.99
1.02
4.30
1.04
1.09
1.07
1.13
10.0
9.5
9.0
8.5
8.0
7.5
7.0
6.5
6.0
5.5
5.0
4.5
4.0
3.5
3.0
2.5
2.0
1.5
1.0
0.5
0.0
f1 (ppm)

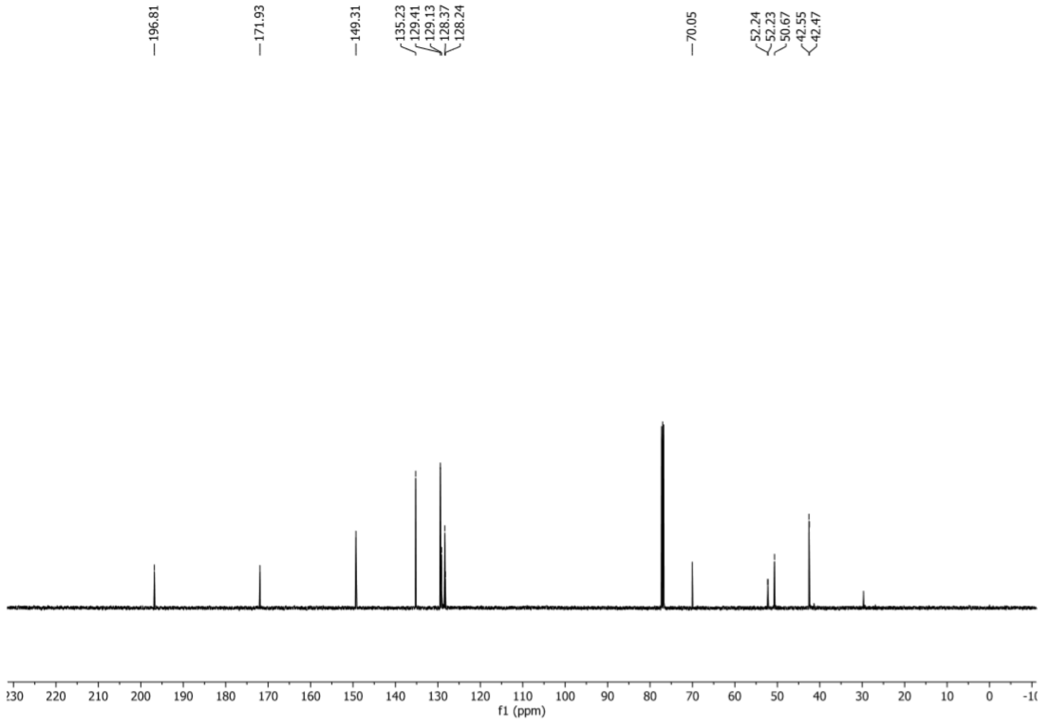

196.81
171.93
149.31
135.23
129.41
129.13
128.37
128.24
70.05
52.24
52.23
50.67
42.55
42.47
220
210
200
190
180
170
160
150
140
130
120
110
100
90
80
70
60
50
40
30
20
10
0
f1 (ppm)

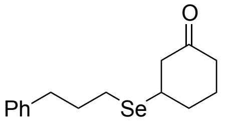
O
Ph
Se

33

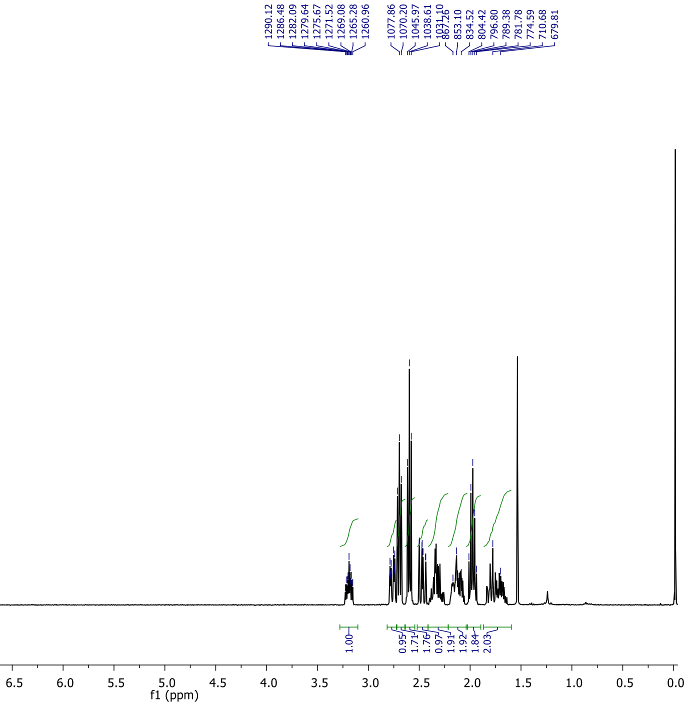
1290.12
1286.48
1282.09
1279.64
1275.67
1271.52
1269.08
1265.28
1260.96
1077.86
1070.20
1045.97
1038.61
853.10
834.52
804.42
796.80
789.38
781.78
774.59
710.68
679.81
4.73
1.00
0.95
1.71
1.76
0.97
1.91
1.92
1.84
2.03
f1 (ppm)

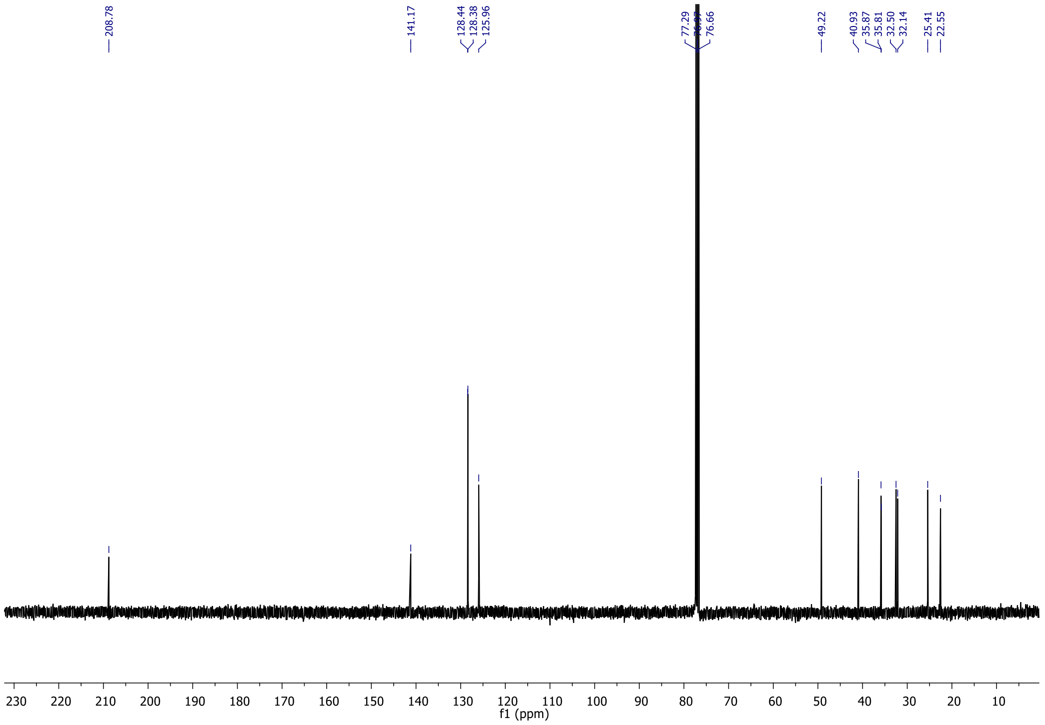
208.78
141.17
128.44
128.38
125.96
77.29
76.66
49.22
40.93
35.87
35.81
32.50
32.14
25.41
22.55
f1 (ppm)

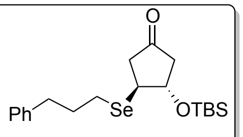
O
Ph
Se
OTBS

34

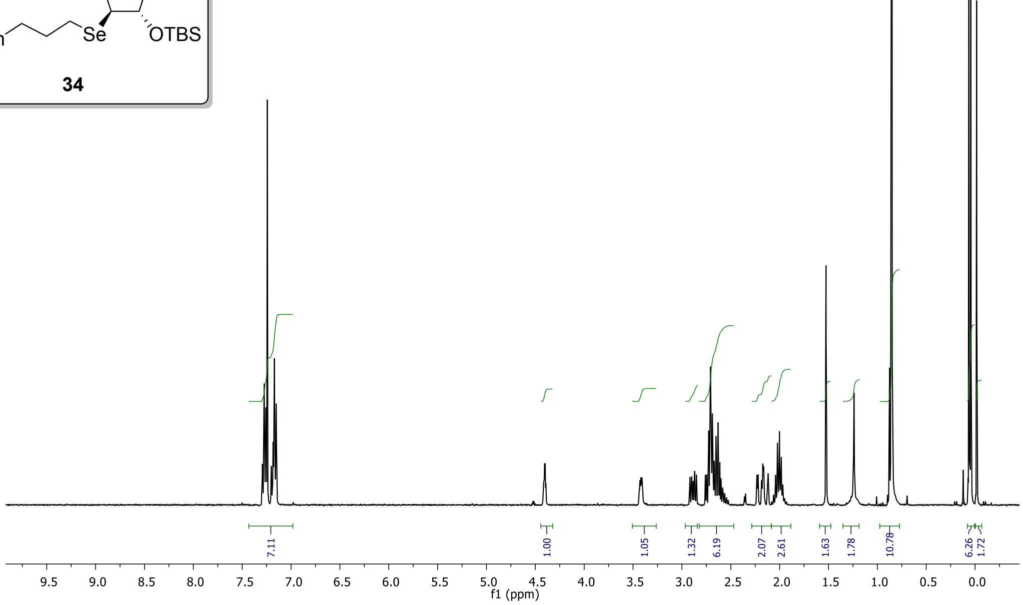
7.11
1.00
1.05
1.32
6.19
2.07
2.61
1.63
1.78
10.78
6.26
1.72
9.5 9.0 8.5 8.0 7.5 7.0 6.5 6.0 5.5 5.0 4.5 4.0 3.5 3.0 2.5 2.0 1.5 1.0 0.5 0.0
f1 (ppm)

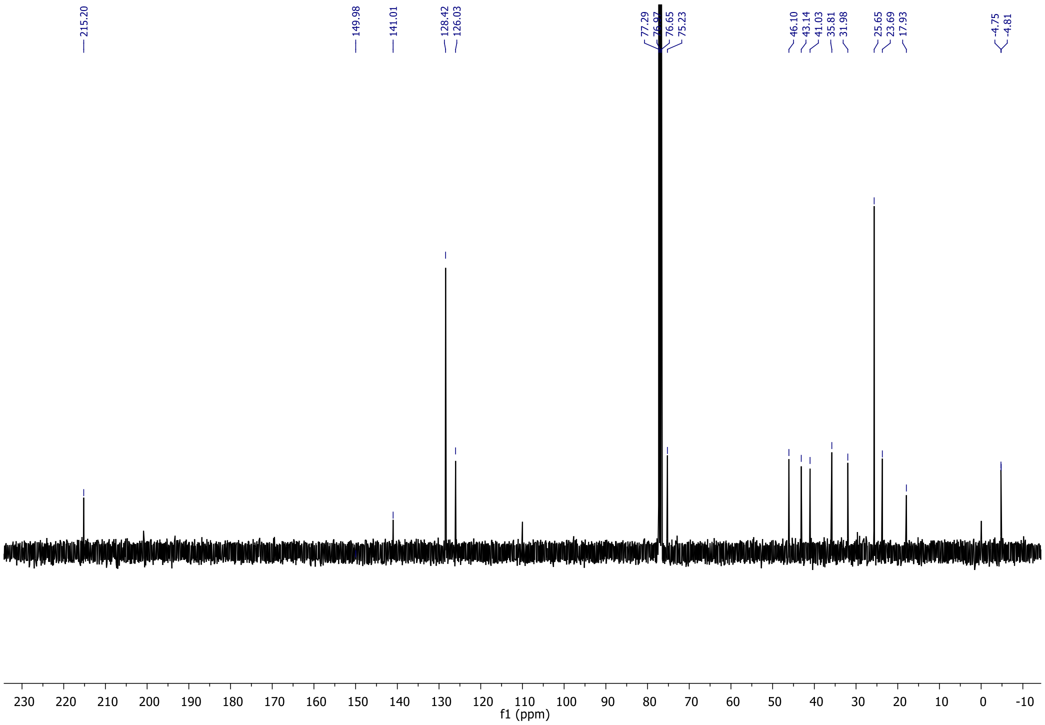
215.20
149.98
141.01
128.42
126.03
77.29
76.97
76.65
75.23
46.10
43.14
41.03
35.81
31.98
25.65
23.69
17.93
-4.75
-4.81
230 220 210 200 190 180 170 160 150 140 130 120 110 100 90 80 70 60 50 40 30 20 10 0 -10
f1 (ppm)

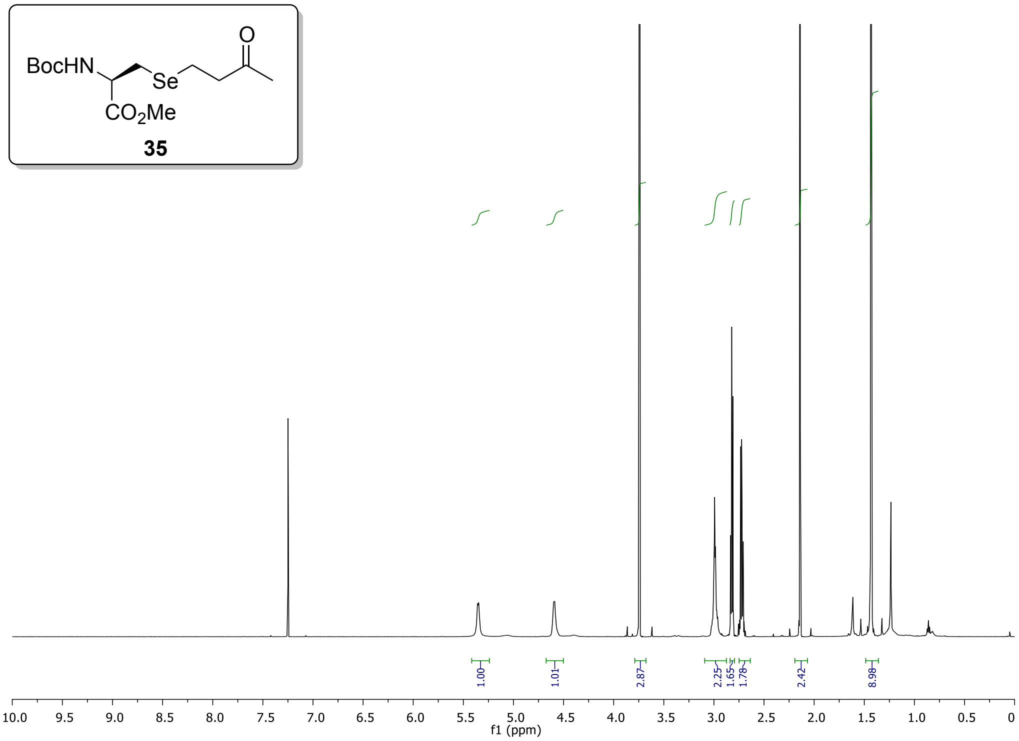

O
BocHN
Se
CO2Me
35
1.00
1.01
2.87
2.25
1.65
1.78
2.42
8.98
f1 (ppm)

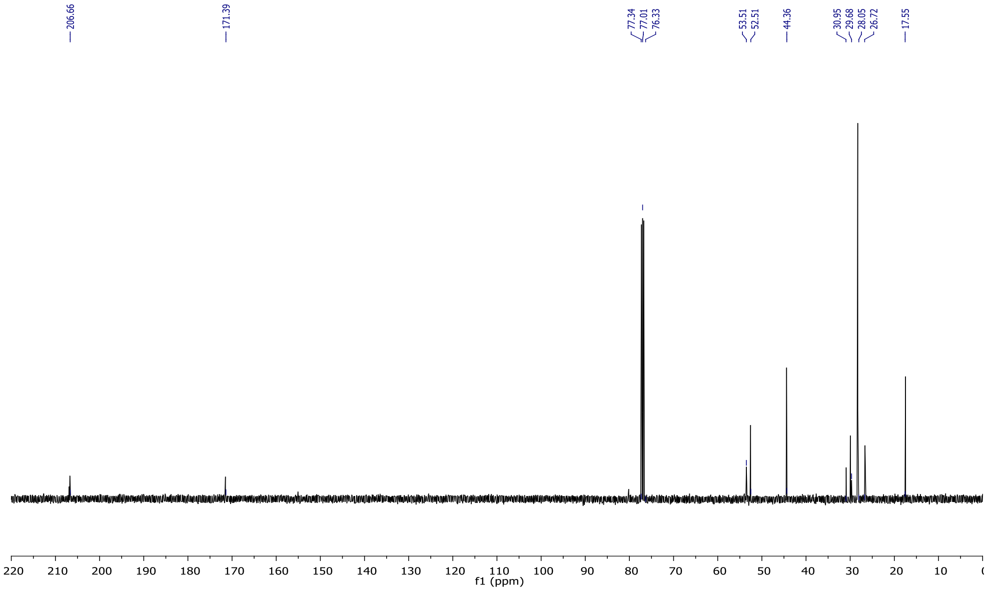

206.66
171.39
77.34
77.01
76.33
53.51
52.51
44.36
30.95
29.68
28.05
26.72
17.55
f1 (ppm)

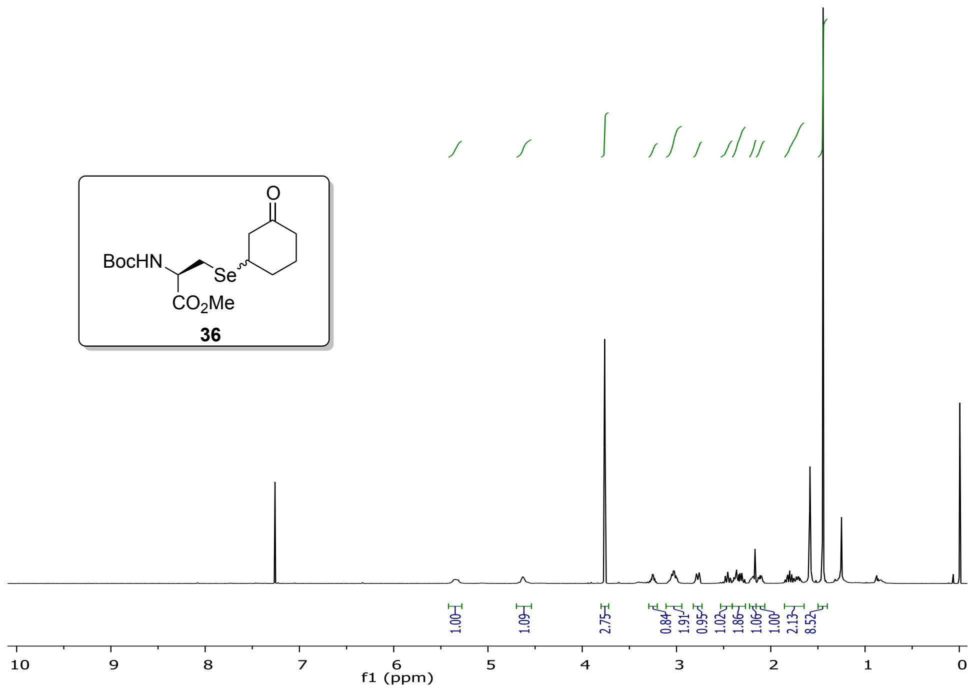
BocHN
Se
CO2Me
O
36
1.00
1.09
2.75
0.84
1.91
0.95
1.02
1.86
1.06
1.00
2.13
8.52
f1 (ppm)

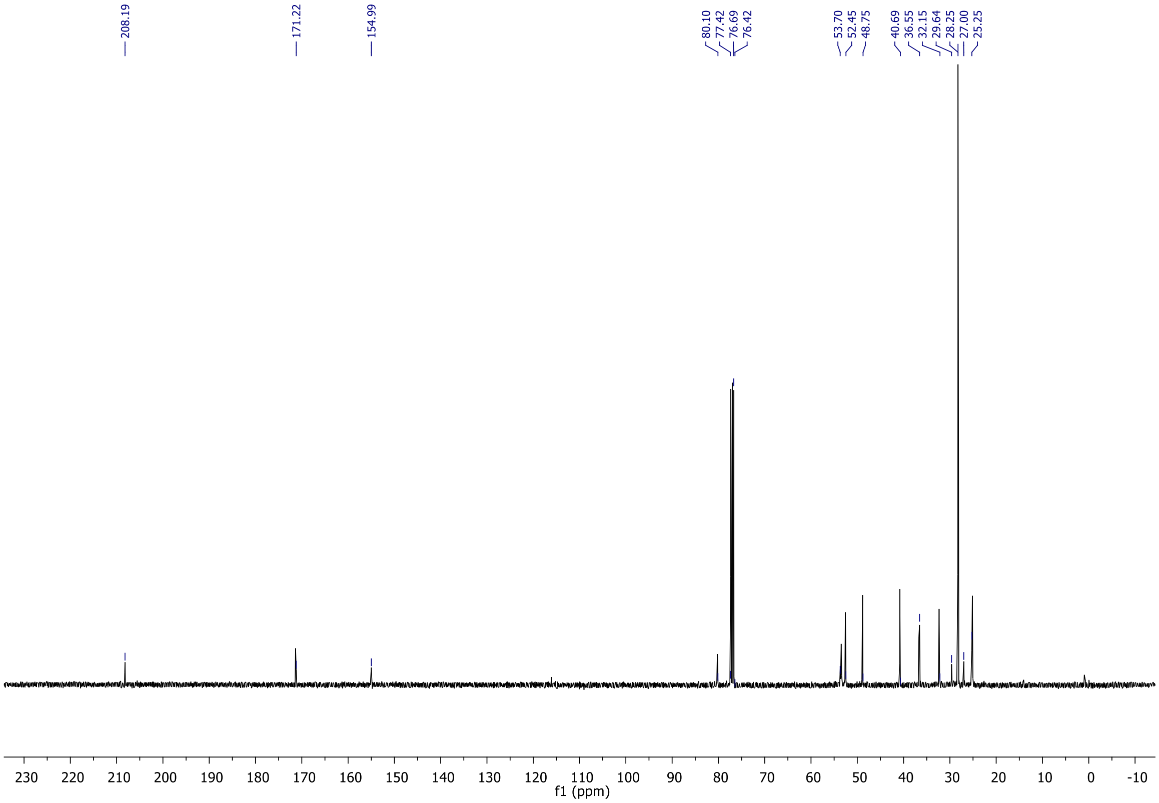
208.19
171.22
154.99
80.10
77.42
76.69
76.42
53.70
52.45
48.75
40.69
36.55
32.15
29.64
28.25
27.00
25.25
f1 (ppm)

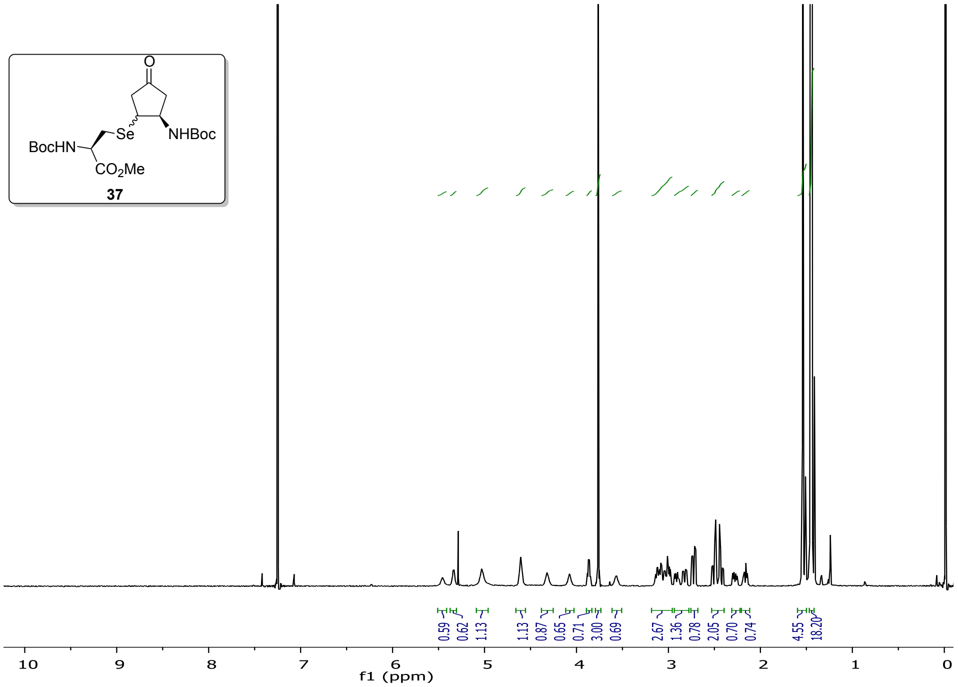
O
Se
NHBoc
BocHN
CO2Me
37
0.59
0.62
1.13
1.13
0.87
0.65
0.71
3.00
0.69
2.67
1.36
0.78
2.05
0.70
0.74
4.55
18.20
10
9
8
7
6
5
4
3
2
1
0
f1 (ppm)

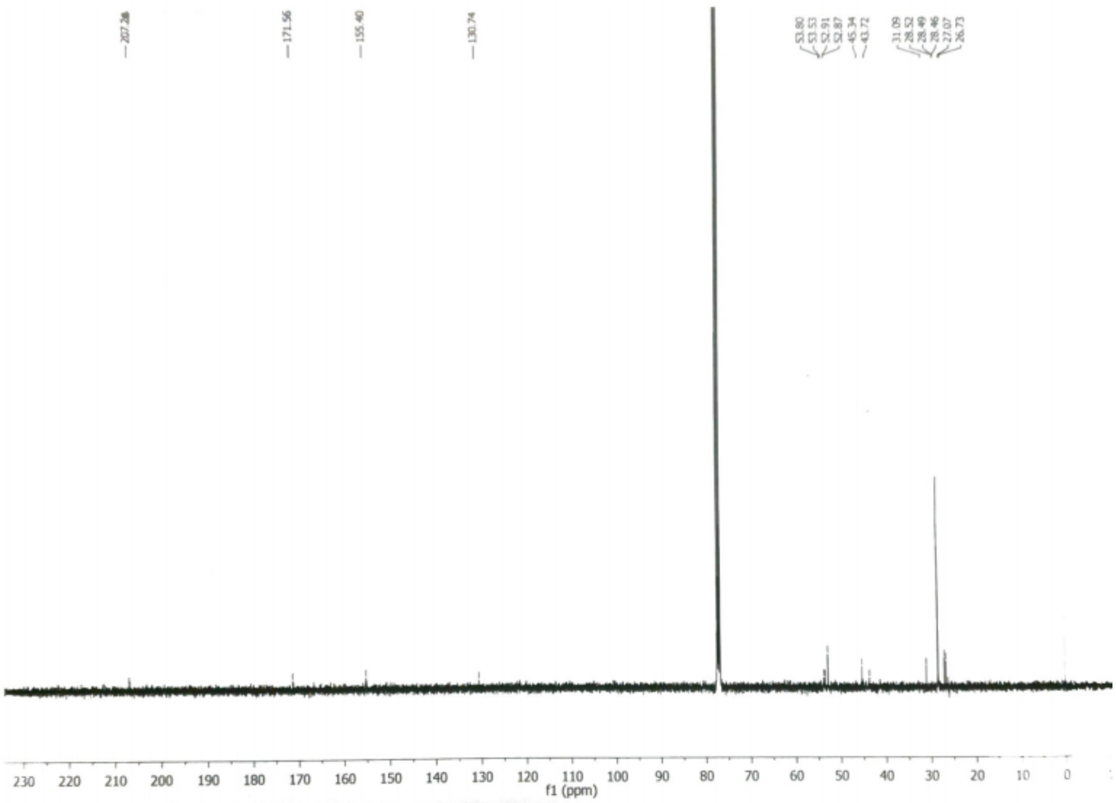
207.28
171.56
155.40
130.74
53.80
53.53
52.91
52.87
45.34
43.72
31.09
28.52
28.49
28.46
27.07
26.73
230
220
210
200
190
180
170
160
150
140
130
120
110
100
90
80
70
60
50
40
30
20
10
0
f1 (ppm)
